# Sustainable spectrofluorimetric determination of berberine in dietary supplements via Erythrosin B Ion-Pair complexation with mechanistic investigation, Box-Behnken optimization, and green chemistry assessment

**DOI:** 10.1038/s41598-026-36903-6

**Published:** 2026-02-03

**Authors:** Humood Al Shmrany, Ali Alqahtani, Taha Alqahtani, Adil Alshehri, Ahmed A. Almrasy

**Affiliations:** 1 Department of Medical Laboratory, College of Applied Medical Sciences, Prince Sattam bin Abdulaziz University, Alkharj, 11942 Saudi Arabia; 2 Department of Pharmacology, College of Pharmacy, King Khalid University, Abha, 62529 Saudi Arabia; 3 Department of Medicine, College of Medicine, King Khalid University, Abha, 62529 Saudi Arabia; 4 Pharmaceutical Analytical Chemistry Department, Faculty of Pharmacy, Al-Azhar University, Cairo, 11751 Egypt

**Keywords:** Berberine, Box-Behnken design, Erythrosin b, Spectrofluorimetric, Green analytical chemistry, Biochemistry, Chemistry

## Abstract

A novel spectrofluorimetric method was developed for berberine determination in dietary supplements based on ion-pair complex formation with Erythrosin B. Spectroscopic characterization of Erythrosin B, berberine, and their complex revealed hypsochromic shift and hypochromic effect in UV-visible absorption upon complexation, with concentration-dependent fluorescence quenching at 555 nm (λ_ex_ = 530 nm). Mechanistic investigations confirmed static quenching through ground-state complex formation, evidenced by decreasing Stern-Volmer constants with increasing temperature (3.68 × 10^5^ to 2.77 × 10^5^ M^− 1^ from 298 to 313 K) and bimolecular quenching rate constants exceeding the diffusion-controlled limit. Thermodynamic analysis indicated spontaneous, exothermic complexation driven by both enthalpic and entropic contributions. Moreover, Job’s method established 1:1 stoichiometry, while PM3 quantum mechanical calculations revealed multiple stabilizing interactions including hydrogen bonding (1.7 Å), electrostatic interaction (3.2 Å), and halogen bonding (3.9 Å). Subsequently, Box-Behnken design optimization elucidated the influence of pH, reagent concentration, buffer volume, and incubation time on fluorescence quenching efficiency and yielded optimal conditions (pH 6.4, Erythrosin B 13.0 µg/mL, buffer volume 1.1 mL, incubation time 6.0 min). The method was then validated according to ICH Q2(R2) guidelines, demonstrating excellent linearity (0.1–3.0 µg/mL, r^2^ = 0.9997), high sensitivity (LOD = 0.032 µg/mL), satisfactory accuracy (99.83%), and precision (RSD < 1.4%). Selectivity studies revealed cross-reactivity with structurally related quaternary alkaloids, rendering the method suitable for standardized berberine supplements but not crude botanical extracts. Furthermore, statistical comparison with HPLC reference methods confirmed method equivalence. Finally, environmental assessment using AGREE (0.75), MoGAPI (78), CaFRI (82), and BAGI (77.5) demonstrated the method superior sustainability through minimal solvent consumption, reduced waste generation, and elimination of toxic reagents, offering a practical and environmentally friendly alternative for routine berberine quality control.

**Supplementary Information:**

The online version contains supplementary material available at 10.1038/s41598-026-36903-6.

## Introduction

Berberine (5,6-dihydro-9,10-dimethoxybenzo[g]-1,3-benzodioxolo[5,6-a]quinolizinium) is a quaternary protoberberine alkaloid widely distributed in various medicinal plants, including *Berberis* species (barberry), *Coptis chinensis* (goldthread), *Hydrastis canadensis* (goldenseal), and *Phellodendron amurense* (Amur cork tree)^1,2^. This natural compound has been utilized in traditional Chinese and Ayurvedic medicine for centuries, and contemporary pharmacological research has substantiated its diverse therapeutic properties^3^. Berberine exhibits significant biological activities including antimicrobial effects against bacterial, fungal, and parasitic pathogens^4,5^, antidiabetic effects through AMPK activation and improved insulin sensitivity^6,7^, hypolipidemic properties via LDL receptor upregulation and PCSK9 inhibition^8,9^, and anti-inflammatory and cardioprotective activities^10,11^. These multifaceted pharmacological properties have driven exponential growth of the global berberine market, with increasing availability of berberine-containing dietary supplements and standardized herbal formulations^12^. However, significant variations in berberine content among commercial products raise concerns regarding quality consistency and consumer safety^13,14^. Consequently, regulatory agencies including the FDA and EMA emphasize the necessity of validated analytical methods for quality assurance, making the development of reliable, sensitive, and cost-effective methods for berberine quantification a critical requirement for quality control and regulatory compliance.

The determination of berberine in pharmaceutical preparations and herbal products has been extensively investigated using various analytical techniques. High-performance liquid chromatography (HPLC) coupled with ultraviolet (UV) or diode array detection remains the most widely adopted method, demonstrating linearities typically ranging from 0.30 to 372.6 µg/mL with limits of detection between 35 and 1500 ng/mL^15–17^. However, HPLC methods require elaborate sample preparation procedures, including solid-phase extraction consuming 3–8 mL of organic solvents per sample, with total analysis times ranging from 15 to 30 min^17,18^. Moreover, the dependence on expensive chromatographic instrumentation, specialized columns, and high-purity mobile phases limits accessibility for routine quality control in resource-constrained laboratories. Alternatively, liquid chromatography-tandem mass spectrometry (LC-MS/MS) provides enhanced selectivity and sensitivity for berberine quantification in complex matrices such as rat plasma^19^. Nevertheless, LC-MS/MS systems necessitate substantial capital investment, rigorous maintenance protocols, and highly trained personnel and generating considerable hazardous waste. Capillary electrophoresis has also been explored as a separation technique for berberine analysis, offering reduced solvent consumption and rapid separation^20^. Despite these advantages, capillary electrophoresis suffers from poor reproducibility, baseline drift, and limited applicability to complex herbal matrices due to matrix-induced signal suppression. In contrast, potentiometric sensors based on ion-selective electrodes offer real-time monitoring capabilities and require minimal sample preparation^21^. However, these sensors suffer from limited selectivity in complex matrices, sensitivity to pH variations and ionic strength, interference from structurally similar alkaloids, requirement for frequent sensor reconditioning, and complex fabrication procedures involving ion-pair formation with reagents such as phosphotungstic acid or sodium tetraphenylborate^21^. Consequently, the development of alternative analytical approaches that combine high sensitivity, operational simplicity, cost-effectiveness, and minimal environmental impact remains a priority in pharmaceutical quality control.

Spectrofluorimetric methods offer distinct advantages over chromatographic techniques, including high sensitivity, operational simplicity, cost-effective instrumentation, and reduced solvent consumption^22–24^. Despite these advantages, spectrofluorimetric methods for berberine determination remain limited and face several practical challenges. Liu et al. developed a spectrofluorimetric method based on supramolecular interaction with β-cyclodextrin dimer^25^. However, this method required precise pH control, extended equilibration times, and relatively expensive cyclodextrin reagents with limited commercial availability. Another study based on cucurbit^7^uril cavity encapsulation was developed for berberine quantification^26^. However, cucurbiturils remain specialty chemicals with restricted availability and complex synthesis procedures. Another study reported a fluorimetric method utilizing silica nanoparticles as probes^27^. Nevertheless, this approach required careful particle size control and surface modification procedures. Additionally, Liang et al. developed another spectrofluorimetric method using DNA-directed silver nanoclusters^28^. However, the method involved complex nanocluster synthesis and required acetic acid medium for fluorescence enhancement. Despite these developments, ion-pair complexation between berberine and anionic fluorescent dyes remains unexplored. Ion-pair formation offers spontaneous electrostatic association without derivatization^29^. Erythrosin B, an anionic xanthene dye, presents favorable characteristics for cationic quaternary alkaloid determination through spontaneous electrostatic interaction. Moreover, xanthene dyes exhibit excitation and emission at longer wavelengths in the visible region, minimizing interference from autofluorescence of biological matrices and co-extracted phytochemicals^30^. Therefore, Erythrosin B represents a promising probe for developing a simple, selective spectrofluorimetric method for berberine determination based on ion-pair complex formation. However, in order to optimize the critical experimental parameters affecting the analytical performance, Design of Experiments approaches such as Box-Behnken design can be employed as a systematic optimization strategy to enhance method robustness and efficiency^31–35^.

In this context, the present study aims to develop and validate a novel spectrofluorimetric method based on ion-pair complex formation between berberine and Erythrosin B for sensitive determination of berberine in commercial formulations. The specific objectives include: (1) comprehensive spectral characterization of Erythrosin B, berberine, and their ion-pair complex using UV-visible absorption and fluorescence spectroscopy; (2) mechanistic investigations including determination of association constant through Stern-Volmer analysis, elucidation of thermodynamic parameters (ΔH°, ΔS°, ΔG°) through temperature dependent studies, establishment of complex stoichiometry using Job’s method, and investigation of binding energies through semiempirical PM3 quantum chemical calculations; (3) systematic optimization of critical experimental parameters including pH, ionic strength, reagent concentration and incubation time using Box-Behnken design; (4) validation of the developed method according to ICH Q2(R2) guidelines; (5) application to commercial berberine formulations with statistical comparison to HPLC reference methods; and finally (6) environmental sustainability assessment using multiple greenness metrics to evaluate the method’s environmental impact and practical applicability. This investigation represents the first report of Erythrosin B-berberine ion-pair complexation for analytical purposes, offering a simple, sensitive, and environmentally conscious alternative for routine berberine quality control.

## Experimental

### Reagents and materials

Berberine chloride (C_20_H_18_ClNO_4_, 98.52% purity) and Erythrosin B disodium salt (C_20_H_6_I_4_Na_2_O_5_, dye content ≥ 95%) were obtained from Sigma-Aldrich (St. Louis, MO, USA). Boric acid, phosphoric acid, acetic acid, and sodium hydroxide, all of analytical reagent grade, were purchased from Piochem Co. (Cairo, Egypt). Commercial berberine dietary supplement capsules (labeled claim: 800 mg berberine per capsule, 60 capsules per container, Enzymedica brand) and berberine hydrochloride capsules (labeled claim: 500 mg berberine HCl per capsule, 90 capsules per container, NOW Foods brand) were purchased from local pharmacies for method application studies. All chemicals were used as received without further purification. Distilled water was used throughout the experiments for solution preparation and dilution.

Britton-Robinson universal buffer solutions were prepared by mixing equimolar concentrations (0.04 M) of boric acid, phosphoric acid, and acetic acid, with pH adjustment achieved by addition of 0.2 M sodium hydroxide solution. A standard berberine chloride solution (100 µg/mL) was prepared by accurately weighing 10.0 mg of berberine chloride and dissolving in distilled water in a 100 mL volumetric flask. An Erythrosin B stock solution (0.01% w/v, equivalent to 100 µg/mL) was prepared by dissolving 10.0 mg of Erythrosin B in 100 mL distilled water. Working solutions were prepared by appropriate dilution with distilled water.

### Instrumentation

Fluorescence measurements were conducted on a Jasco FP-6200 spectrofluorometer (Jasco Inc., Easton, MD, USA) with excitation and emission slit widths of 10 nm. Ultraviolet-visible absorption spectra were recorded using a Shimadzu UV-1800 double-beam spectrophotometer (Shimadzu Corporation, Kyoto, Japan) with 1 cm path length quartz cuvettes. Solution pH was measured using a Jenway 3510 benchtop pH meter (Jenway Ltd., Felsted, UK) with accuracy of ± 0.01 pH. Accurate weighing of chemicals was performed using a Sartorius BP 211D analytical balance (Sartorius AG, Göttingen, Germany) with readability of 0.01 mg. Sample sonication was carried out using an Elmasonic S30H ultrasonic bath (Elma Schmidbauer GmbH, Singen, Germany).

### Experimental design and optimization

Preliminary investigations were conducted to identify the critical variables affecting the fluorescence quenching of the berberine-Erythrosin B ion-pair complex and to determine appropriate factor ranges for optimization. Based on these initial screening studies, a four-factor, three-level Box-Behnken design was employed to systematically optimize the experimental parameters. The independent variables investigated were solution pH (X_1_: 3.0–8.0), Erythrosin B concentration (X_2_: 5.0–15.0 µg/mL), Britton-Robinson buffer volume (X_3_: 0.5–1.5 mL), and incubation time (X_4_: 4–10 min). The fluorescence quenching ratio (F_0_/F) at 555 nm (excitation 530 nm) was selected as the response variable, where F_0_ represents the fluorescence intensity of Erythrosin B alone and F represents the fluorescence intensity in the presence of berberine. The experimental design matrix consisted of 27 runs, including 24 factorial points and 3 center point replicates for estimation of pure error. Design-Expert software (Version 11, Stat-Ease Inc., Minneapolis, MN, USA) was used for experimental design generation, statistical analysis, and response surface modeling.

For each experimental run, the general procedure was as follows: an aliquot of berberine working solution equivalent to 3.0 µg/mL final concentration was transferred into a 10 mL volumetric flask. The specified volume of Britton-Robinson buffer at the designated pH was added, followed by the required volume of Erythrosin B solution to achieve the target concentration according to the design matrix. The solution was diluted to volume with distilled water, mixed thoroughly, and allowed to stand for the specified incubation time at room temperature (25 ± 1 °C). For F_0_ measurement, a blank solution containing the same concentration of Erythrosin B without berberine was prepared under identical conditions. Fluorescence intensity was measured at λ_em_ = 555 nm with λ_ex_ = 530 nm for both the berberine-containing solution (F) and the blank solution (F_0_), and the quenching ratio (F_0_/F) was calculated. All measurements were performed in triplicate.

The experimental data were analyzed using multiple regression analysis to fit a second-order polynomial equation:

$$Y =\beta_0+\sum \beta_iX_i+\sum \beta_{ii}X_{i}^{2}+\sum \beta_{ij}X_iX_j$$

where Y is the predicted response (F_0_/F), β₀ is the intercept, β_i_ are linear coefficients, β_i__i_ are quadratic coefficients, and β_i_ⱼ are interaction coefficients. Backward elimination procedure was applied to remove statistically insignificant terms (*p* > 0.05) from the full model, retaining only significant factors and interactions to obtain a reduced model with improved predictive capability. Analysis of variance (ANOVA) was performed to evaluate the statistical significance of the final model and individual terms. The adequacy of the model was assessed through coefficient of determination (R^2^), adjusted R^2^, predicted R^2^, adequate precision, and residual analysis. Three-dimensional response surface plots and two-dimensional contour plots were generated to visualize the relationship between factors and response, and to identify optimal conditions.

### General analytical procedure and calibration curve construction

Under the optimized experimental conditions established by the Box-Behnken design, the general analytical procedure for berberine determination was as follows: Into a series of 10 mL volumetric flasks, accurate aliquots of berberine working solution were transferred to obtain final concentrations ranging from 0.1 to 3.0 µg/mL. To each flask, 1.1 mL of Britton-Robinson buffer (pH 6.4) was added, followed by 1.3 mL of Erythrosin B solution (100 µg/mL stock) to achieve a final concentration of 13.0 µg/mL. The solutions were diluted to volume with distilled water and mixed thoroughly by gentle inversion. After standing for 6.0 min at room temperature (25 ± 1 °C) to ensure complete ion-pair formation, fluorescence intensity was measured at emission wavelength 555 nm with excitation at 530 nm using slit widths of 10 nm for both excitation and emission. A reagent blank containing all components except berberine was prepared similarly and measured to obtain F_0_. The fluorescence quenching ratio (F_0_/F) was calculated for each berberine concentration, where F represents the fluorescence intensity in the presence of berberine. The calibration curve was constructed by plotting the fluorescence quenching ratio (F_0_/F) against berberine concentration. Each concentration point on the calibration curve was measured in triplicate (*n* = 3), and the mean fluorescence quenching ratio (F_0_/F) was calculated. Linear regression analysis was performed to establish the relationship between fluorescence quenching and berberine concentration. The limit of detection (LOD) and limit of quantification (LOQ) were calculated as 3.3σ/S and 10σ/S, respectively, where σ is the standard deviation of the response for the blank (*n* = 10) and S is the slope of the calibration curve.

### Mechanistic investigation and quantum mechanical calculations

Fluorescence quenching studies were conducted at three temperatures (298, 303, and 313 K) to investigate the interaction mechanism. For each temperature, a series of solutions were prepared in 10 mL volumetric flasks by adding 1.1 mL Britton-Robinson buffer (pH 6.4) and 1.3 mL Erythrosin B solution (100 µg/mL) to achieve a final concentration of 13.0 µg/mL. Varying volumes of berberine working solution were added to obtain final concentrations ranging from 0.5 to 3.0 µg/mL. Solutions were diluted to volume with distilled water, mixed thoroughly, and equilibrated for 6 min at the designated temperature. Fluorescence intensity was measured at λex = 530 nm and λem = 555 nm. The Stern-Volmer equation (F_0_/F = 1 + Ksv[Q]) was applied to analyze the fluorescence quenching behavior, where F0 represents the fluorescence intensity of Erythrosin B in the absence of berberine (quencher), F is the fluorescence intensity in the presence of berberine at concentration [Q], and Ksv is the Stern-Volmer quenching constant (M^− 1^) that quantifies the quenching efficiency. Linear plots of F0/F versus berberine concentration [Q] at each temperature yielded Ksv values from the slopes. Association constants (Ka) were obtained from the modified Stern-Volmer equation using double reciprocal plots of F_0_/(F_0_-F) against 1/[Q], providing independent verification of the Ksv values. Thermodynamic parameters (ΔH°, ΔS°, ΔG°) were determined from the van’t Hoff equation (ln Ka = -ΔH°/RT + ΔS°/R) by plotting ln Ka versus 1/T.

The stoichiometric ratio was determined using Job’s method of continuous variation. Stock solutions of Erythrosin B and berberine were prepared at 6.00 × 10^− 5^ M concentration in distilled water. A series of 10 mL solutions were prepared by mixing different volumes of the two stock solutions according to the mole fraction sequence while maintaining a constant total molar concentration of 6.00 × 10^− 6^ M. Each solution was allowed to equilibrate for 6 min at 25 °C. Fluorescence intensity was measured at λex = 530 nm and λem = 555 nm. The change in fluorescence intensity (ΔF = F_0_ - F) was plotted against the mole fraction of berberine to determine the stoichiometric ratio from the maximum point.

Quantum mechanical calculations were performed using Gaussian 09 W software with the PM3 semiempirical method. Initial structures of Erythrosin B (charge − 2), berberine (charge + 1), and their 1:1 complex (charge − 1) were constructed using GaussView 6.0. Full geometry optimization was performed without symmetry constraints. Binding energy was calculated as ΔE_binding_ = E(complex) - [E(EB) + E(Berb)]. Molecular properties including dipole moments and polarizabilities were extracted from output files.

### Analysis of commercial formulations

The validated method was applied to the determination of berberine in commercial dietary supplement capsules. Five capsules from each product were emptied, and the combined contents were weighed accurately and mixed thoroughly to ensure homogeneity. A portion of the mixed powder equivalent to approximately 10 mg of berberine was accurately weighed and transferred into a 100 mL volumetric flask. The powder was dissolved in 50 mL distilled water with sonication for 15 min to ensure complete extraction. The solution was cooled to room temperature, diluted to volume with distilled water, and filtered through 0.45 μm membrane filter. An appropriate aliquot of the filtrate was further diluted to obtain a final concentration within the calibration range. The analysis was performed following the general analytical procedure described in Sect. "General analytical procedure and calibration curve construction". Each sample was analyzed in triplicate, and the berberine content was calculated from the calibration curve. Results compared with the label claim to calculate percentage recovery. For method comparison, the same sample solutions were analyzed using a validated HPLC reference method^17^. Statistical evaluation was performed using Student’s t-test and F-test at 95% confidence level to assess significant differences between the proposed fluorescence method and the HPLC method. Additionally, interval hypothesis testing was conducted to evaluate method equivalence within acceptable bias limits.

## Results and discussion

### Spectroscopic characterization of erythrosin B, Berberine, and their complex

**Fig. 1:**
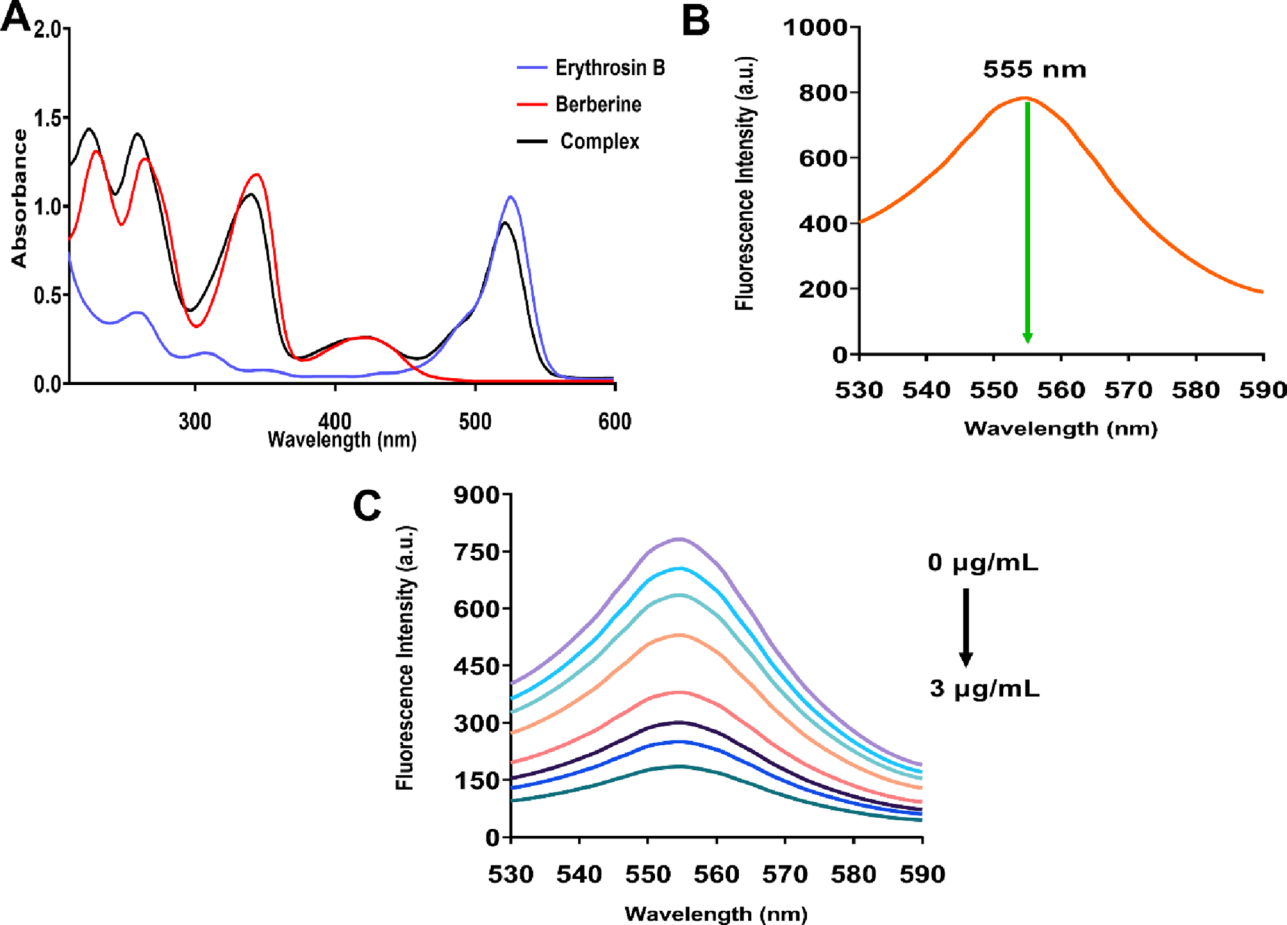
Spectroscopic characterization of Erythrosin B, berberine, and their ion-pair complex. (**A**) UV-visible absorption spectra showing Erythrosin B (blue line), berberine (red line), and the complex (black line), with hypsochromic shift and hypochromic effect upon complexation. (**B**) Fluorescence emission spectrum of Erythrosin B with excitation at 530 nm and maximum emission at 555 nm. (**C**) Concentration-dependent fluorescence quenching of Erythrosin B upon incremental addition of berberine (0 to 3 µg/mL).

The UV-visible absorption spectrum of Erythrosin B in aqueous solution exhibited a characteristic intense absorption band at λ_max_ = 530 nm (Fig. 1A), which is attributed to the π→π* transition of the xanthene chromophore (Fig. S1A). The extended conjugated system of the xanthene nucleus, combined with the electron-withdrawing effect of the four iodine substituents at positions 2, 4, 5, and 7, results in bathochromic shift of the absorption maximum into the visible region and enhanced molar absorptivity. In contrast, berberine displayed multiple absorption bands at approximately 230, 265, 345, and 420 nm (Fig. 1A), consistent with literature reports for isoquinoline alkaloids. The absorption band at 345 nm corresponds to the π→π* transition of the benzene ring, while the band at 420 nm arises from the extended conjugation across the protoberberine skeleton involving the quaternary nitrogen and the dioxole ring system (Fig. S1B). Upon formation of the ion-pair complex between Erythrosin B and berberine, significant spectral changes were observed in the UV-visible region. The characteristic absorption maximum of Erythrosin B underwent a hypsochromic shift of 5 nm from 530 nm to 525 nm, accompanied by a pronounced hypochromic effect manifested as decreased absorbance intensity (Fig. 1A). These spectral perturbations provide direct evidence for ground state complex formation. The hypsochromic shift suggests increased energy separation between ground and excited states upon complexation, while the hypochromic effect indicates restricted mobility and altered electronic distribution in the complex compared to free Erythrosin B.

The fluorescence properties of Erythrosin B were investigated to establish the analytical signal for berberine determination. Erythrosin B exhibited maximum excitation at 530 nm and maximum emission at 555 nm, yielding a Stokes shift of 25 nm (Fig. 1B). This relatively small Stokes shift is characteristic of xanthene dyes and reflects minimal structural reorganization between ground and excited states. The intense fluorescence emission of Erythrosin B originates from the rigid planar xanthene framework, which restricts non-radiative decay pathways. Interestingly, despite the presence of four heavy iodine atoms that typically promote intersystem crossing and fluorescence quenching through the internal heavy atom effect, Erythrosin B maintains appreciable fluorescence quantum yield due to the stabilization provided by the extended aromatic conjugation. Upon incremental addition of berberine (0 to 3 µg/mL), systematic and concentration-dependent quenching of Erythrosin B fluorescence was observed at λ_em_ = 555 nm (Fig. 1C). The fluorescence intensity decreased progressively with increasing berberine concentration while maintaining the spectral profile shape, indicating that the emission mechanism remained unchanged and quenching occurred without altering the excited state properties of the fluorophore. Based on these spectroscopic characteristics and the observed fluorescence response, excitation and emission wavelengths of 530 nm and 555 nm, respectively, were selected for all subsequent analytical measurements.

### Mechanistic investigations

#### Stern-Volmer analysis and thermodynamics studies

To elucidate the fluorescence quenching mechanism between Erythrosin B and berberine, several potential quenching pathways were systematically evaluated, including dynamic quenching, static quenching, inner filter effect, and Förster resonance energy transfer (FRET). The inner filter effect was excluded based on the negligible absorbance of berberine at the excitation (530 nm) and emission (555 nm) wavelengths used for Erythrosin B measurements (Fig. 1A). FRET was also ruled out due to the extremely weak fluorescence emission of berberine in aqueous solution at the studied wavelengths, insufficient spectral overlap between Erythrosin B emission and berberine absorption, and the absence of characteristic donor-acceptor spectral features in the UV-visible data.

**Fig. 2:**
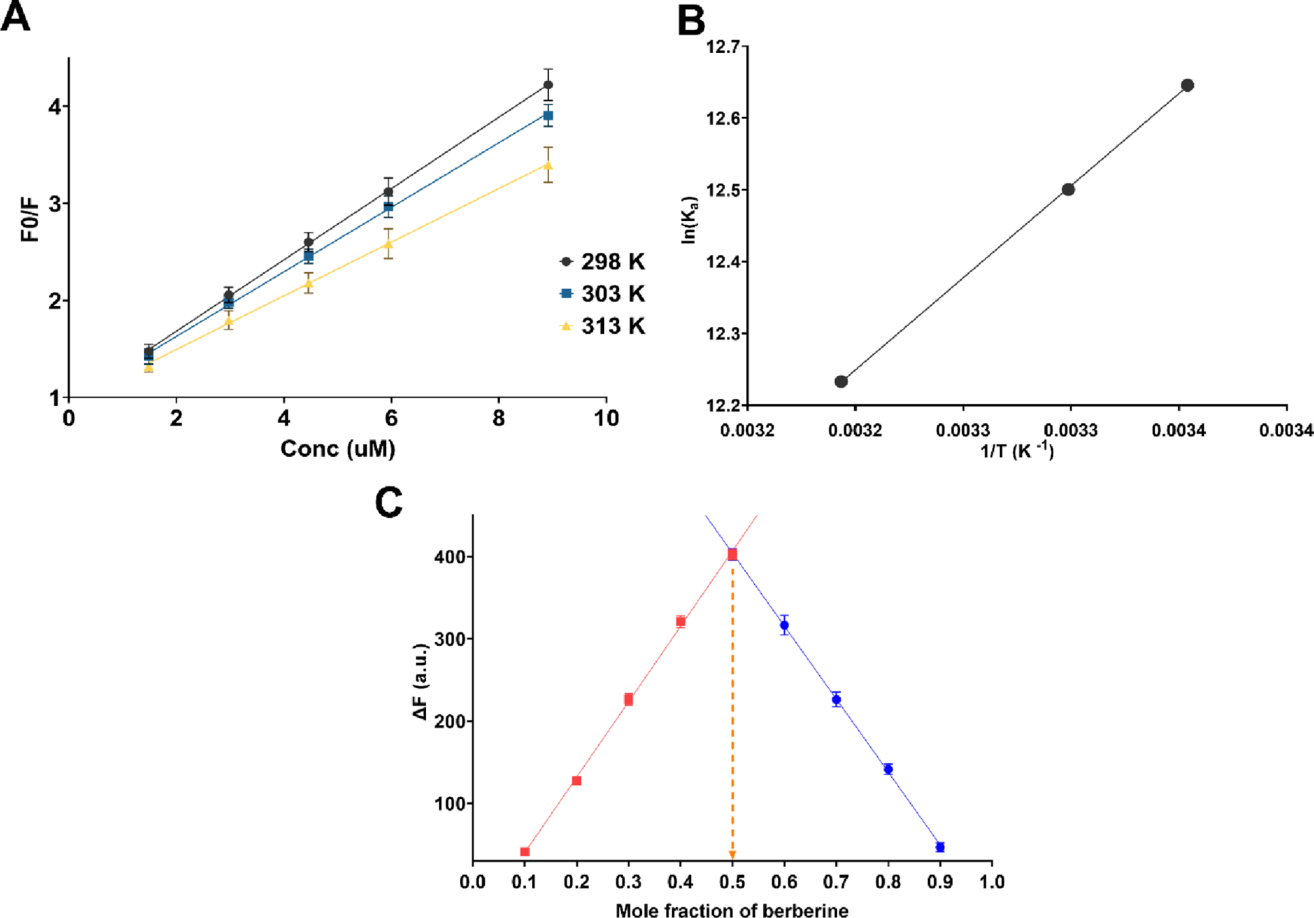
Mechanistic investigation of Erythrosin B-berberine interaction. (**A**) Stern-Volmer plots at three temperatures (298, 303, and 313 K) showing linear relationships between F_0_/F and berberine concentration with decreasing slopes at elevated temperatures. (**B**) van’t Hoff plot of ln Ka versus 1/T for determination of thermodynamic parameters. (**C**) Job’s plot showing the change in fluorescence intensity (ΔF) versus mole fraction of berberine, with maximum at 0.5 confirming 1:1 stoichiometry.

The distinction between static and dynamic quenching was established through temperature-dependent fluorescence studies conducted at 298, 303, and 313 K. Dynamic quenching arises from collisional encounters between excited-state fluorophore and quencher molecules, with quenching efficiency increasing at elevated temperatures. Conversely, static quenching involves formation of a non-fluorescent ground-state complex, with complex stability decreasing at higher temperatures due to thermal dissociation. The Stern-Volmer plots obtained at three temperatures exhibited linear relationships between F_0_/F and berberine concentration (Fig. 2A). The Stern-Volmer quenching constants (Ksv) showed a clear decreasing trend with increasing temperature: 3.68 × 10^5^ M^− 1^ at 298 K, 3.33 × 10^5^ M^− 1^ at 303 K, and 2.77 × 10^5^ M^− 1^ at 313 K (Table S1). This negative temperature dependence is characteristic of static quenching and confirms ground-state complex formation.

To further verify the static mechanism, the bimolecular quenching rate constant (Kq) was calculated using Kq = Ksv/τ₀, where τ₀ represents the fluorescence lifetime previously reported for Erythrosin B (89 ps). The calculated Kq values (4.13 × 10^13^ M^− 1^ s^− 1^ at 298 K, 3.74 × 10^13^ M^− 1^ s^− 1^ at 303 K, and 3.11 × 10^13^ M^− 1^ s^− 1^ at 313 K) were approximately three orders of magnitude higher than the maximum diffusion-controlled quenching rate constant (2.0 × 10^10^ M^− 1^ s^− 1^), providing definitive evidence for static quenching. The association constants (Ka) determined from Stern-Volmer analysis followed the same temperature-dependent trend: 3.10 × 10^5^ M^− 1^ at 298 K, 2.74 × 10^5^ M^− 1^ at 303 K, and 2.05 × 10^5^ M^− 1^ at 313 K (Table S1), indicating strong electrostatic association between the dianionic Erythrosin B and monocationic berberine.

The thermodynamic parameters for the ion-pair complexation were determined from the temperature dependence of the association constants using the van’t Hoff equation: ln Ka = -ΔH°/RT + ΔS°/R. The van’t Hoff plot of ln Ka versus 1/T exhibited excellent linearity (Fig. 2B), from which the enthalpy change (ΔH°) and entropy change (ΔS°) were calculated from the slope and intercept, respectively. The obtained values were ΔH° = -21.36 kJ mol^− 1^ and ΔS° = 33.51 J mol^− 1^ K^− 1^ (Table S1). The negative enthalpy change confirms that the complexation process is exothermic, consistent with electrostatic ion-pair formation being energetically favorable. The positive entropy change suggests increased disorder upon complex formation, which can be attributed to the release of water molecules from the hydration shells of both Erythrosin B and berberine during ion-pair association. The Gibbs free energy changes (ΔG°) calculated at each temperature using the relationship ΔG° = ΔH° - TΔS° were − 31.35 kJ mol^− 1^ at 298 K, -31.56 kJ mol^− 1^ at 303 K, and − 31.85 kJ mol^− 1^ at 313 K (Table S1). The negative ΔG° values at all temperatures confirm the spontaneous nature of the complexation process. The slight increase in the magnitude of ΔG° with temperature, despite the decrease in Ka, reflects the entropic contribution becoming more significant at elevated temperatures. Collectively, the thermodynamic parameters indicate that the Erythrosin B-berberine interaction is driven by both enthalpy (electrostatic attraction) and entropy (desolvation), with the process being spontaneous and exothermic under the studied analytical conditions. The stoichiometric ratio of the Erythrosin B-berberine complex was determined using Job’s method of continuous variation. Solutions containing varying mole fractions of berberine and Erythrosin B were prepared while maintaining a constant total molar concentration of 6.00 × 10^− 6^ M. The change in fluorescence intensity (ΔF = F_0_ - F) was plotted against the mole fraction of berberine (Fig. 2C). The Job’s plot exhibited a symmetrical profile with a distinct maximum at a mole fraction of 0.5, unambiguously confirming a 1:1 stoichiometric ratio for the ion-pair complex.

#### Quantum mechanical studies

**Fig. 3:**
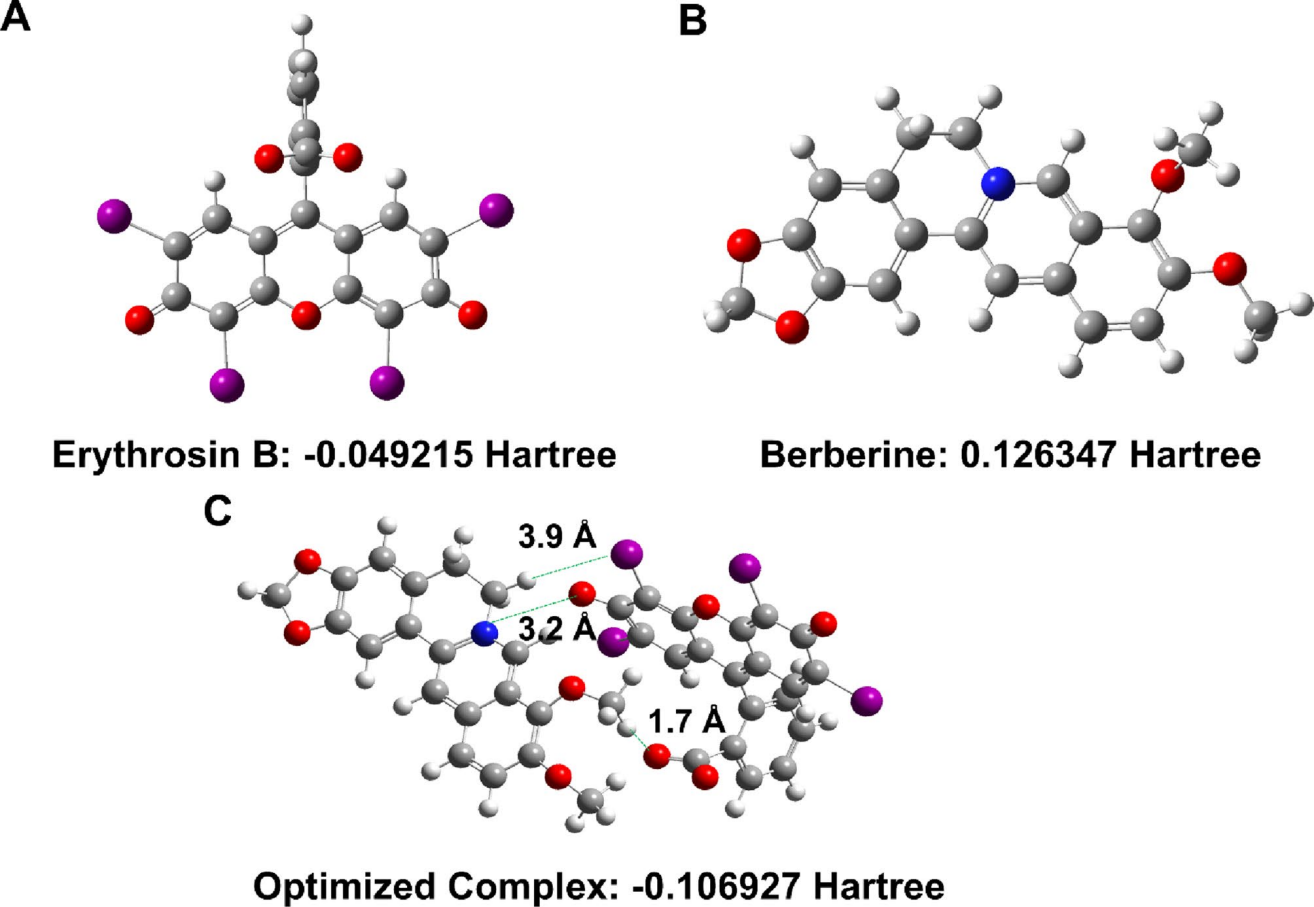
PM3-optimized structures of (**A**) Erythrosin B, (**B**) berberine, and (**C**) their 1:1 ion-pair complex showing three stabilizing interactions: hydrogen bond (1.7 Å), electrostatic interaction (3.2 Å), and halogen bond (3.9 Å).

To gain molecular-level insights into the ion-pair complex formation and validate the experimental observations, semiempirical quantum mechanical calculations were performed using the PM3 method. Full geometry optimizations were conducted for Erythrosin B (charge − 2), berberine (charge + 1), and their 1:1 ion-pair complex without symmetry constraints. The optimized structures are presented in Fig. 3, and the calculated molecular properties are summarized in Table 1. The optimization convergence was confirmed by the low root-mean-square (RMS) gradient values, indicating well-converged geometries at true energy minima (Table 1). The optimized structure of Erythrosin B displayed a planar xanthene core with iodine substituents oriented perpendicular to the aromatic plane, while berberine exhibited the characteristic nearly planar protoberberine skeleton with slight deviation from planarity due to the dioxole ring system (Fig. 3A & B). The calculated dipole moments were 12.77 D for Erythrosin B and 2.83 D for berberine, reflecting their respective charged states and asymmetric charge distributions (Table 1). The polarizability values (357.99 and 230.39 a.u. for Erythrosin B and berberine, respectively) indicate the electronic response to external electric fields, with the larger, more extended π-system of Erythrosin B exhibiting greater polarizability.

**Table 1:** Quantum mechanical properties of erythrosin B, berberine, and their ion-pair complex calculated using PM3 semiempirical method.

Property	Erythrosin B	Berberine	Complex	Interpretation
Energy (Hartree)	-0.049215	0.126347	-0.106927	Complex more stable than sum of parts
Charge	-2	+ 1	-1	Charge neutralization
Dipole (D)	12.77	2.83	25.55	NOT additive - charge redistribution
Polarizability α (a.u.)	357.99	230.39	572.29	Nearly additive (588.38 expected)
Hyperpolarizability β (a.u.)	5154.95	2012.98	5400.59	Less than sum - charge delocalization
RMS Gradient	0.000013	0.000003	0.000006	All well-converged

The optimized geometry of the 1:1 ion-pair complex revealed multiple stabilizing interactions contributing to complex formation (Fig. 3C). Three distinct interaction sites were identified: a strong hydrogen bond (1.7 Å) between the carboxyl group of Erythrosin B and a hydrogen atom of the methoxy group of berberine, a primary electrostatic interaction (3.2 Å) between the quaternary nitrogen of berberine and the hydroxyl group of Erythrosin B, and a halogen bond (3.9 Å) between an iodine atom and a hydrogen atom (Fig. 3C). These multiple interaction points suggest that complex stabilization arises from a combination of electrostatic attraction, hydrogen bonding, and weaker dispersive forces. The binding energy was determined from the optimized energies: -0.106927 Hartree for the complex versus − 0.049215 Hartree for Erythrosin B and + 0.126347 Hartree for berberine. The resulting binding energy of -0.184059 Hartree indicates highly favorable complex formation. However, this value should be interpreted cautiously as PM3 methods tend to overestimate binding energies for charged species due to limitations in treating electrostatic interactions and lack of solvation effects in the gas-phase calculations. Nevertheless, the negative binding energy qualitatively confirms the experimental observation of spontaneous complex formation (ΔG° = -31.35 kJ mol⁻¹).

The electronic properties of the complex provided further insights into the nature of the interaction. The dipole moment of the complex (25.55 D) was significantly non-additive compared to the sum of individual dipole moments (15.60 D), indicating substantial charge redistribution and electronic reorganization upon complexation (Table 1). This charge redistribution is consistent with the observed spectral changes in UV-visible absorption (hypsochromic shift and hypochromic effect) and supports the formation of a ground-state complex with altered electronic structure. The polarizability of the complex (572.29 a.u.) was nearly additive (expected 588.38 a.u.), suggesting that the overall electronic response to external fields is largely preserved, with minimal mutual polarization between the components. Interestingly, the hyperpolarizability (β) of the complex (5400.59 a.u.) was less than the sum of individual values (7167.93 a.u.), indicating reduced non-linear optical response due to charge delocalization and symmetry considerations in the complex geometry (Table 1). Collectively, the quantum mechanical calculations support the electrostatic ion-pair mechanism proposed based on experimental data, reveal multiple interaction sites contributing to complex stability, and provide molecular-level rationalization for the observed spectroscopic and thermodynamic behavior.

### Optimization of analytical parameters using Box-Behnken design

A four-factor, three-level Box-Behnken design was employed to systematically optimize the experimental conditions for maximum fluorescence quenching efficiency. Based on preliminary screening studies, the investigated factors and their ranges were: solution pH (A: 3.0–8.0), Erythrosin B concentration (B: 5.0–15.0 µg/mL), Britton-Robinson buffer volume (C: 0.5–1.5 mL), and incubation time (D: 4–10 min). The fluorescence quenching ratio (F_0_/F) at λ_em_ = 555 nm was selected as the response variable. The experimental design consisted of 27 runs, including 24 factorial points and 3 center point replicates for pure error estimation (Table S2). The observed F_0_/F responses ranged from 1.13 to 4.22, demonstrating substantial variation across the experimental domain and confirming the need for systematic optimization. A second-order polynomial model was fitted to the experimental data, and backward elimination was applied to remove statistically insignificant terms (*p* > 0.05), yielding a reduced model containing only significant main effects, quadratic effects, and interaction terms.

Analysis of variance (ANOVA) revealed that the reduced model was highly significant (F = 68.41, *p* < 0.0001), indicating adequate representation of the relationship between factors and response (Table 2). The model exhibited excellent fit statistics: R² = 0.9682, adjusted R² = 0.9540, and predicted R² = 0.9322 (Table S3). The difference between adjusted R² and predicted R² was less than 0.20, confirming satisfactory predictive capability. Adequate precision, which measures the signal-to-noise ratio, was 26.38, substantially exceeding the minimum threshold of 4, thereby validating the model’s utility for navigating the design space. The lack of fit was not significant (F = 1.42, *p* = 0.4918), indicating that the model adequately describes the experimental data without systematic error. Among the individual factors, pH (A) exhibited the highest F-value (68.02, *p* < 0.0001), followed by Erythrosin B concentration (B: F = 67.12, *p* < 0.0001) and buffer volume (C: F = 21.17, *p* = 0.0002) (Table 2). Notably, incubation time (D) and all interaction terms involving this factor were statistically insignificant and were eliminated from the model. The quadratic terms A², B², and C² were all highly significant (*p* < 0.0001), with A² showing the largest effect (F = 286.87), indicating strong curvature in the pH response. Two interaction terms were significant: AB (pH × Erythrosin B, F = 40.17, *p* < 0.0001) and BC (Erythrosin B × buffer volume, F = 9.50, *p* = 0.0064). The final reduced model equation expressed in coded factors was:

**Table 2:** Analysis of variance (ANOVA) for the reduced Box-Behnken design model.

Source	Sum of Squares	df	Mean Square	F-value	*p*-value	
Model	20.14	8	2.52	68.41	< 0.0001	Significant
A-pH	2.50	1	2.50	68.02	< 0.0001	
B-Erythrosin B	2.47	1	2.47	67.12	< 0.0001	
C-Buffer Volume	0.7793	1	0.7793	21.17	0.0002	
AB	1.48	1	1.48	40.17	< 0.0001	
BC	0.3498	1	0.3498	9.50	0.0064	
A²	10.56	1	10.56	286.87	< 0.0001	
B²	1.68	1	1.68	45.72	< 0.0001	
C²	4.69	1	4.69	127.36	< 0.0001	
Residual	0.6625	18	0.0368			
Lack of Fit	0.6088	16	0.0380	1.42	0.4918	Not significant
Pure Error	0.0538	2	0.0269			
Cor Total	20.81	26				

F_0_/F = 4.046 + 0.457 A + 0.454B + 0.255 C + 0.608AB + 0.296BC − 1.327 A² − 0.530B² − 0.884 C².

Diagnostic plots confirmed model adequacy and absence of outliers (Fig. S2). The normal probability plot of residuals showed points closely aligned along the diagonal line, indicating normal distribution of residuals (Fig. S2A). The predicted versus actual plot demonstrated strong agreement between model predictions and experimental observations, with points clustering near the 45° line (Fig. S2B). Residuals versus run number showed random scatter without systematic patterns (Fig. S3A), confirming independence of observations. The leverage plot revealed that all experimental points had leverage values below the threshold limit, indicating no influential outliers (Fig. S3B).

**Fig. 4:**
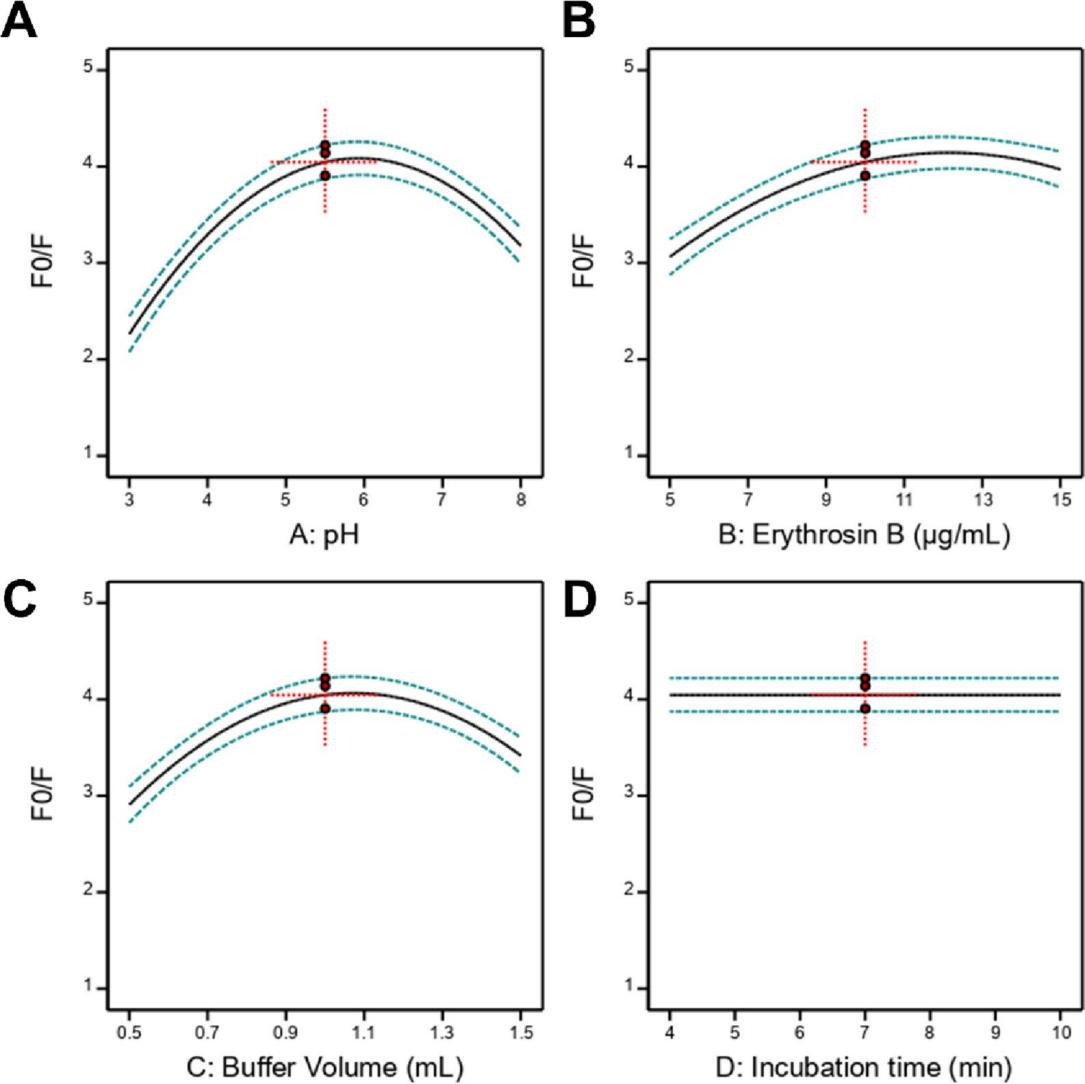
Individual factor effects from Box-Behnken design on fluorescence quenching ratio (F_0_/F). (**A**) Effect of pH showing parabolic relationship with optimum at pH 6.4. (**B**) Effect of Erythrosin B concentration with optimum at 13.0 µg/mL. (**C**) Effect of buffer volume with optimum at 1.1 mL. (**D**) Effect of incubation time showing minimal influence across the studied range.

The effects of individual factors and their interactions on fluorescence quenching were visualized through response surface and contour plots. The individual factor effect plots revealed that pH exhibited a parabolic relationship with F_0_/F, reaching maximum response at approximately pH 6.4 (Fig. 4A). This behavior reflects the ionization states of both Erythrosin B and berberine, with optimal complex formation occurring at intermediate pH where both molecules possess appropriate charges for electrostatic association. Erythrosin B concentration showed a similar quadratic profile, with optimal response at approximately 13.0 µg/mL (Fig. 4B). Higher concentrations led to decreased response due to self-quenching phenomena and inner filter effects. Buffer volume displayed quadratic dependence with optimum near 1.1 mL (Fig. 4C), balancing adequate buffering capacity against dilution effects. Incubation time had minimal effect on response across the studied range (Fig. 4D), indicating rapid complex formation kinetics, consistent with the instantaneous nature of electrostatic ion-pair association.

**Fig. 5:**
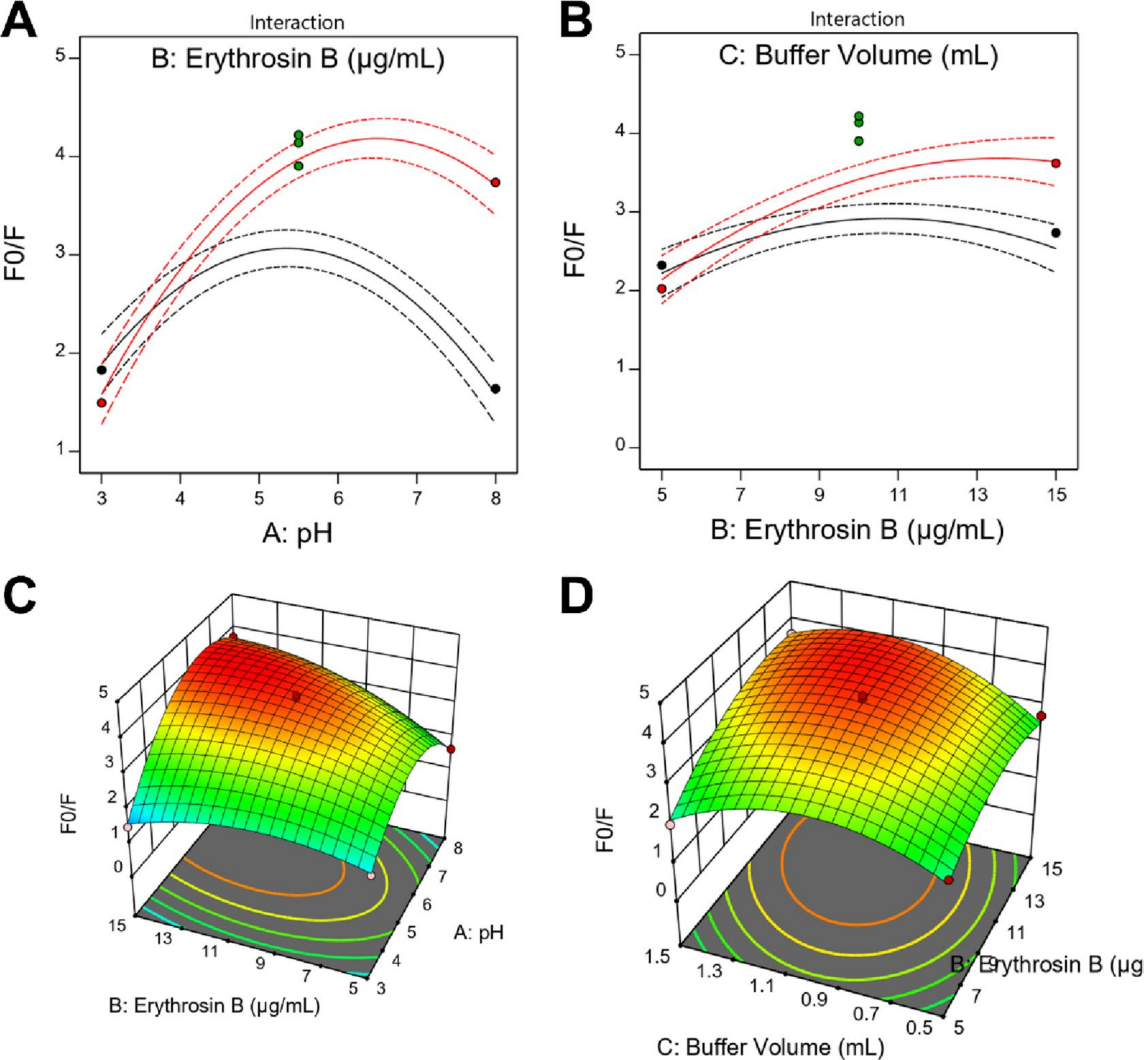
Interaction effects and response surface analysis. (**A**) Two-dimensional interaction plot for pH and Erythrosin B concentration. (**B**) Two-dimensional interaction plot for Erythrosin B concentration and buffer volume. (**C**) Three-dimensional response surface plot showing the combined effect of pH and Erythrosin B concentration on F_0_/F. (**D**) Three-dimensional response surface plot illustrating the interaction between Erythrosin B concentration and buffer volume.

The interaction between pH and Erythrosin B concentration (AB) was visualized through 2D interaction and 3D response surface plots (Fig. 5A and C). At low pH values (< 4.0), increasing Erythrosin B concentration produced minimal improvement in F_0_/F, whereas at optimal pH (5.5–6.5), Erythrosin B concentration exerted pronounced effects. This synergistic interaction suggests that both factors must be simultaneously optimized to achieve maximum quenching efficiency. Similarly, the interaction between Erythrosin B concentration and buffer volume (BC) demonstrated that optimal buffer volume shifted depending on reagent concentration (Fig. 5B and D). At higher Erythrosin B concentrations (> 12 µg/mL), larger buffer volumes were required to maintain optimal ionic strength and prevent aggregation.

Numerical optimization was performed using the desirability function approach to identify conditions maximizing F_0_/F (Fig. S4). The optimal conditions were determined as: pH 6.43, Erythrosin B concentration 12.99 µg/mL, buffer volume 1.06 mL, and incubation time 6.35 min, with a predicted maximum F_0_/F of 4.29 (Fig. S5). For practical implementation, these values were rounded to pH 6.4, Erythrosin B 13.0 µg/mL, buffer volume 1.1 mL, and incubation time 6.0 min. Experimental validation at these conditions yielded F_0_/F = 4.25 ± 0.08 (*n* = 5), demonstrating excellent agreement with the predicted value (0.93% deviation) and confirming model validity. The overlay plot (Fig. S4) identified a yellow feasible region where all optimization criteria were simultaneously satisfied, providing operational flexibility around the optimal point. These optimized conditions were adopted for all subsequent analytical method validation and sample analysis experiments.

### Method validation according to ICH Q2(R2) guidelines

The developed spectrofluorimetric method was comprehensively validated following ICH Q2(R2) guidelines to ensure reliability, reproducibility, and fitness for purpose^36^. Under the optimized experimental conditions (pH 6.4, Erythrosin B 13.0 µg/mL, buffer volume 1.1 mL, incubation time 6.0 min), a calibration curve was constructed by plotting the fluorescence quenching ratio (F_0_/F) against berberine concentration. The method exhibited excellent linearity over the concentration range of 0.1–3.0 µg/mL, with a regression equation of F_0_/F = 1.0829[Berb] + 0.9677 and correlation coefficient (r^2^) of 0.9997 (Table 3). The high r^2^ value indicates strong linear correlation between response and concentration, fulfilling ICH requirements. The limit of detection (LOD) and limit of quantification (LOQ), calculated as 3.3σ/S and 10σ/S respectively (where σ is the standard deviation of blank responses, *n* = 10, and S is the slope), were 0.032 µg/mL and 0.096 µg/mL, respectively (Table 3). These sensitivity parameters demonstrate the method’s capability to detect and quantify berberine at trace levels, comparing favorably with reported HPLC methods (LOD: 0.035–1.5 µg/mL) while eliminating the need for chromatographic separation and expensive instrumentation.

**Table 3:** Validation parameters for the developed spectrofluorimetric method according to ICH Q2(R2) guidelines.

Parameters		Berberine
Excitation wavelength (nm)		530
Emission wavelength (nm)		555
Linearity range (µg/mL)		0.1-3.0
Slope		1.0829
Intercept		0.9677
Correlation coefficient (r^2^)		0.9997
LOD (µg/mL)		0.032
LOQ (µg/mL)		0.096
Accuracy (%R) ^a^		99.83 ± 0.982
Repeatability precision (%RSD) ^b^		0.984
Intermediate precision (%RSD) ^c^		1.318
Robustness (%R)	Buffer (pH)	99.76 ± 0.834
	Reagent Conc. (µg/mL)	99.59 ± 1.613
	Reaction time (min)	101.77 ± 0.811

^a^ Average of 9 determinations (3 concentrations repeated 3 times).

^b^ % RSD of 9 determinations (3 concentrations repeated 3 times) measured on the same day.

^c^ % RSD of 9 determinations (3 concentrations repeated 3 times) measured in the three consecutive days.

Method accuracy was evaluated through recovery studies at three concentration levels (low, medium, high) within the linear range (Table S4). The mean recovery was 99.83 ± 0.982% (*n* = 9), demonstrating excellent accuracy with bias less than 1% from theoretical values (Table 3). Precision was assessed at two levels: repeatability (intra-day precision) and intermediate precision (inter-day precision). Repeatability, determined by analyzing three different concentrations in triplicate on the same day under identical conditions (Table S4), yielded a relative standard deviation (RSD) of 0.984%. Intermediate precision, evaluated by repeating the same analysis on three consecutive days (Table S4), resulted in RSD of 1.318% (Table 3). Both precision parameters were well below the ICH acceptance criterion of RSD ≤ 2%, confirming the method’s reproducibility. Robustness was investigated by introducing deliberate variations in critical experimental parameters: pH (6.4 ± 0.2), Erythrosin B concentration (13.0 ± 1.0 µg/mL), and incubation time (6.0 ± 1.0 min). The recoveries remained within acceptable limits: 99.76 ± 0.834% for pH variation, 99.59 ± 1.613% for reagent concentration variation, and 101.77 ± 0.811% for time variation (Table 3), demonstrating that minor operational fluctuations do not significantly impact method performance. The stability of the berberine-Erythrosin B ion-pair complex was investigated by monitoring the fluorescence quenching ratio (F0/F) at regular time intervals after complex formation. The complex exhibited excellent stability at room temperature (25 ± 1 °C), with the fluorescence signal remaining constant (RSD < 2%) for at least 2 h. Measurements performed at 0, 30, 60, 90, and 120 min after mixing showed variations of less than 1.5%, confirming that the complex remains stable throughout the analytical measurement period. This stability provides an adequate time window for routine analytical measurements and demonstrates the practical suitability of the method for batch analysis without concerns about time-dependent signal degradation.

Selectivity was comprehensively evaluated by testing potential interfering substances commonly encountered in berberine formulations. Nine pharmaceutical excipients typically present in capsule and tablet dosage forms were investigated, including lactose monohydrate, microcrystalline cellulose, magnesium stearate, starch, gelatin, titanium dioxide, silicon dioxide, polyvinylpyrrolidone, and sodium stearyl fumarate (Table S5). Each excipient was added at concentrations 10-fold higher than the active ingredient. The quenching efficiency (QE%) remained statistically unchanged (67.65–68.45%) compared to the control (67.96%), with RSD values below 2.5%, indicating no interference from these matrix components. This finding confirms the method’s suitability for analyzing commercial formulations without elaborate sample cleanup procedures. Subsequently, cross-reactivity with structurally related compounds was investigated to define the method’s selectivity profile. Seven phytochemicals were evaluated, including quaternary alkaloids and common plant phenolics (Table S6). Neutral and anionic phenolic compounds (quercetin, rutin, chlorogenic acid) exhibited no interference, with QE% values (68.03–68.17%) equivalent to berberine alone. However, structurally related quaternary protoberberine alkaloids (palmatine, coptisine) and benzophenanthridine alkaloids (sanguinarine) exhibited significant positive interference, increasing the quenching efficiency by 32–40% (Table S6). This cross-reactivity is attributed to the ability of these cationic alkaloids to form ion-pairs with Erythrosin B at the working pH of 6.4, possessing similar quaternary ammonium functional groups and appropriate lipophilicity for electrostatic complexation. The observed interference follows the order: sanguinarine (40%) > palmatine (36%) > coptisine (32%), correlating with their respective charge densities and structural planarity. Consequently, the method measures total quaternary alkaloid content rather than being strictly selective for berberine. This characteristic renders the method suitable for quality control of standardized berberine supplements where berberine constitutes the predominant quaternary alkaloid (> 95% purity), but not appropriate for crude botanical extracts containing multiple quaternary alkaloids without prior separation. For applications requiring absolute specificity, preliminary chromatographic separation or the use of orthogonal HPLC methods would be necessary.

### Analysis of commercial formulations and method comparison

The validated spectrofluorimetric method was applied to determine berberine content in two commercially available dietary supplement products with labeled claims of 800 mg and 500 mg berberine per capsule. Analysis using the developed method yielded berberine contents of 100.94 ± 0.972% (*n* = 5) for the first product and 97.54 ± 0.978% (*n* = 5) for the second product (Table 4). Both formulations demonstrated good agreement with label claims, with deviations within acceptable pharmaceutical limits (90–110% of labeled amount), confirming adequate manufacturing quality control and label accuracy. The low relative standard deviations (< 1%) for both products further demonstrated the method’s precision when applied to real sample matrices containing excipients.

**Table 4:** Analysis of commercial Berberine formulations and statistical comparison with HPLC reference method.

Formulation	Method	Mean ^a^	SD	t-test (2.306) ^b^	*P* value	F-value (6.338) b	*P* value	θ_Λ_^χ^	θ_Υ_^χ^
Berberine Enzymedica	Developed method	100.94	0.97	0.278	0.789	1.727	0.61	-1.46	1.85
	Reported method	100.74	1.28						
Berberine NOW Foods	Developed method	97.54	0.98	0.032	0.975	1.577	0.669	-1.64	1.6
	Reported method	97.56	1.23						

^a^ Average of five determinations.

^b^ The values in parenthesis are tabulated values of “t “and “F” at (P = 0.05).

^c^ Bias of ± 2% is acceptable.

To establish method equivalence with established reference procedures, the same sample solutions were analyzed using a validated HPLC method, and results were compared using three complementary statistical approaches. Student’s t-test was employed to evaluate whether significant differences existed between mean values obtained by the two methods. The calculated t-values (0.278 for product 1 and 0.032 for product 2) were substantially lower than the critical value (tcrit = 2.306 at 95% confidence level), with corresponding p-values of 0.789 and 0.975, respectively (Table 4). These results indicate no statistically significant difference between methods. The F-test was applied to compare precision between methods. The calculated F-values (1.727 for product 1 and 1.577 for product 2) were below the critical value (Fcrit = 6.338 at 95% confidence level), with p-values of 0.610 and 0.669, demonstrating comparable precision (Table 4). Additionally, interval hypothesis testing was performed to assess method equivalence within predefined acceptance limits of ± 2% bias. The 90% confidence intervals for the mean differences between methods were calculated, yielding θ_L_ = -1.455% and θ_U_ = 1.854% for product 1, and θ_L_ = -1.641% and θ_U_ = 1.596% for product 2 (Table 4). Since both confidence intervals fell entirely within the ± 2% acceptance range, statistical equivalence was confirmed according to interval hypothesis testing criteria. Collectively, these statistical evaluations demonstrate that the developed spectrofluorimetric method produces results equivalent to the HPLC reference method for standardized berberine formulations, while offering advantages of simplified analytical procedure, reduced analysis time, lower solvent consumption, and elimination of expensive chromatographic instrumentation.

### Green chemistry and sustainability assessment

The environmental sustainability and practical applicability of the developed spectrofluorimetric method were comprehensively evaluated using four complementary assessment tools: AGREE (Analytical GREEnness Metric)^37^, MoGAPI (Modified Green Analytical Procedure Index)^38^, CaFRI (Carbon Footprint Reduction Index)^39^, and BAGI (Blue Applicability Grade Index)^40^. These metrics provide holistic evaluation encompassing greenness, carbon footprint, and practical applicability, aligning with the principles of sustainable analytical chemistry.

**Fig. 6:**
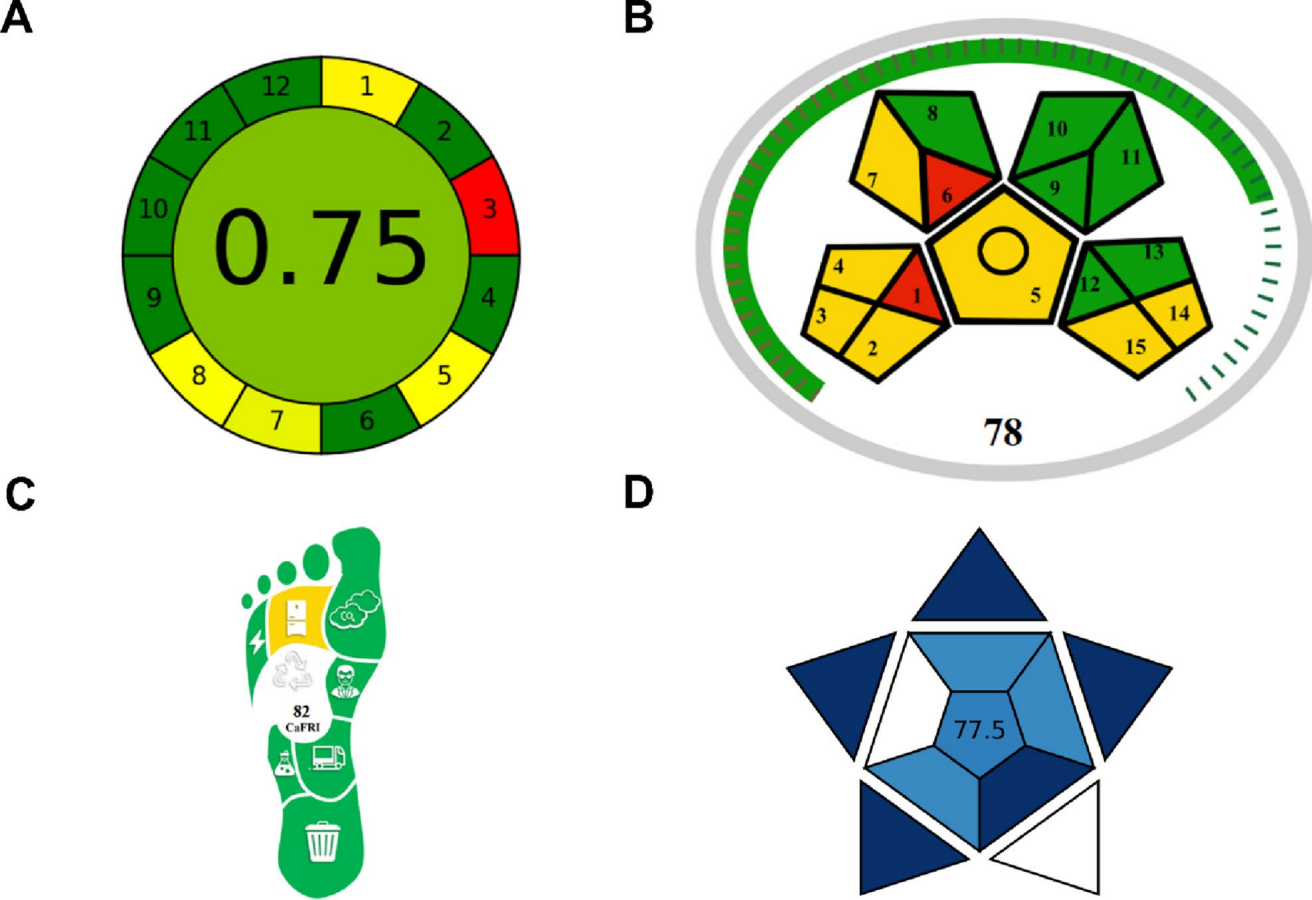
Green chemistry and sustainability assessment. (**A**) AGREE pictogram showing overall score of 0.75 with color-coded evaluation of 12 green analytical chemistry principles. (**B**) MoGAPI pictogram displaying score of 78 for green sample preparation and analytical procedures. (**C**) CaFRI carbon footprint assessment pictogram with score of 82. (**D**) BAGI pictogram showing practical applicability score of 77.5.

The AGREE metric, which assesses adherence to the 12 principles of Green Analytical Chemistry, yielded a score of 0.75 out of 1.0 (Fig. 6A). This score falls within the acceptable green range and reflects favorable performance across multiple green chemistry principles. The method achieved excellent scores for minimal sample size (principle 2), integration of analytical processes (principle 4), avoidance of derivatization (principle 6), minimal energy consumption (principle 9), use of renewable reagents (principle 10), elimination of toxic reagents (principle 11), and operator safety (principle 12). Moderate performance was observed for sample treatment (principle 1) due to the requirement for aqueous extraction and filtration, automation (principle 5) reflecting manual operation, waste generation (principle 7) due to aqueous waste production, and multi-analyte capability (principle 8) as the method determines a single analyte. The primary limitation was in situ measurements (principle 3), as laboratory-based fluorescence instrumentation is required. Despite these constraints, the overall AGREE score demonstrates that the method incorporates most green chemistry principles effectively, particularly those related to reagent toxicity elimination, energy minimization, and operator safety.

The MoGAPI assessment, which focuses on green sample preparation and analytical procedures, yielded a score of 78 out of 100 (Fig. 6B), indicating good environmental performance. The method received favorable scores for employing simple sample preparation procedures, using green solvents (water as primary solvent), generating minimal waste, and implementing appropriate waste treatment. The CaFRI metric, which specifically evaluates carbon footprint and greenhouse gas emissions, produced a score of 82 out of 100 (Fig. 6C), demonstrating excellent performance in minimizing environmental impact. The high CaFRI score is attributed to minimal electrical power consumption, low carbon emission factor, storage under normal conditions without refrigeration, high sample throughput, single-analyst operation, minimal waste generation, use of reagents with low hazard classifications, and minimal organic solvent consumption. These characteristics collectively demonstrate the method’s low carbon footprint and reduced contribution to greenhouse gas emissions compared to conventional chromatographic techniques requiring energy-intensive instrumentation, controlled temperature environments, and larger volumes of organic mobile phases.

The BAGI metric evaluates practical applicability and operational feasibility, yielding a score of 77.5 out of 100 (Fig. 6D), which substantially exceeds the recommended threshold of 60 and confirms high practical utility. The method achieved excellent performance for quantitative analysis capability, simple and commercially available instrumentation, high-throughput simultaneous sample preparation, low-cost procedures, common commercially available reagents, no preconcentration requirement, and small sample amount. Overall, the four sustainability metrics demonstrate that the developed spectrofluorimetric method represents a greener and more sustainable alternative to conventional HPLC methods for berberine determination. The method’s primary environmental advantages include elimination of toxic organic solvents, reduced energy consumption, minimal waste generation, ambient temperature operation, simplified sample preparation, and use of benign aqueous reagents. These characteristics align with current trends toward sustainable analytical methodologies and contribute to reduced environmental impact, lower operational costs, and improved laboratory safety, making the method particularly suitable for routine quality control in resource-limited settings and supporting the transition toward environmentally friendly pharmaceutical analysis.

### Comprehensive comparison with reported fluorescence-based methods for berberine determination

The extensive literature on berberine fluorescence detection encompasses diverse methodological approaches, ranging from ion-association methods to sophisticated nanomaterial-based sensors (Table S7). Most critically relevant to the present work are two studies that previously reported berberine-erythrosin interactions using fundamentally different detection principles. Liu and Feng developed a resonance Rayleigh scattering (RRS) method employing erythrosin B and other acidic xanthene dyes in the presence of cinchona alkaloids^41^. Their approach measured RRS intensity at λex = 370 nm and λem = 452 nm at pH 4.0–5.0, achieving a linear range of 0.04–1.2 µM with LOD of 1.4 × 10⁻⁸ M (approximately 5.2 ng/mL) and 10-minute analysis time for pharmaceutical tablets. Sakai and Hirose investigated mixed ternary ion-associate formation (1:1:1 stoichiometry) between xanthene dyes (eosin or tetraiodofluorescein), cinchona-alkaloids, and quaternary ammonium ions including berberine^42^. Their method employed UV-visible absorbance measurements at 536–543 nm at pH 8.1 following extraction of the bulky ternary associate into 1,2-dichloroethane, achieving molar absorptivity of approximately 1 × 10⁵ L mol⁻¹ cm⁻¹ over the linear range of 0.25–1.5 µM (approximately 93–560 ng/mL for berberine). While these studies established that berberine forms ion-associates with erythrosin and other xanthene dyes, they employed entirely different detection principles—RRS and UV-visible absorption—and did not explore the fluorescence quenching phenomenon that forms the basis of the present analytical method. More importantly, neither study provided mechanistic investigation through temperature-dependent Stern-Volmer analysis, thermodynamic characterization, stoichiometric determination via Job’s method, quantum mechanical calculations to identify binding sites and interactions, systematic DoE-based optimization, comprehensive ICH-compliant validation, or environmental sustainability assessment. The Sakai method’s requirement for organic solvent extraction further contradicts modern green chemistry principles, while the Liu method’s reliance on cinchona alkaloids and RRS detection adds complexity and requires specialized equipment.

Supramolecular and host-guest chemistry approaches have emerged as attractive alternatives for berberine detection, primarily exploiting fluorescence enhancement through restriction of intramolecular motion or cavity encapsulation. Recent cucurbit[n]uril-based methods have demonstrated considerable promise, with the ns-Q^10^-based supramolecular assembly achieving detection through aggregation-induced emission with application to human serum samples^43^, and the CB^6^-based assembly providing remarkably rapid response within 7 s in environmental water^44^. These systems typically achieve detection limits in the low micromolar range (0.15–4.38 µM) with emission wavelengths around 500–570 nm. Despite their analytical performance, cucurbiturils present significant practical barriers as specialty macrocyclic compounds requiring multi-step synthesis, offering limited commercial availability, and commanding premium prices that make them impractical for routine pharmaceutical quality control laboratories. Traditional β-cyclodextrin inclusion complexes provide more accessible alternatives but demand precise pH control and prolonged equilibration times exceeding 20 min, reducing analytical throughput.

Nanomaterial-based fluorescence methods have dominated recent berberine detection research, leveraging diverse nanosystems to achieve exceptional sensitivity. Quantum dot-based approaches, particularly CdTe quantum dots capped with thioglycolic acid, have demonstrated detection limits as low as 6.0 × 10⁻⁹ mol/L through static fluorescence quenching mechanisms^45^. Metal nanocluster systems have shown remarkable versatility, with gold nanoclusters exhibiting exceptionally large Stokes shifts (237 nm) and achieving detection limits of 7.5 × 10⁻⁸ mol/L through combined inner filter effect and aggregation mechanisms^46^. Advanced silver nanocluster platforms, including DNA-templated AgNCs providing ratiometric detection at 10 nM^47^, chitosan/SiO₂-coated AgNCs achieving 30.8 nM detection^48^, and plasmon-enhanced fluorescence sensors based on Ag nanocubes reaching 87.3 nM^49^, have demonstrated the potential of plasmonic enhancement and energy transfer mechanisms. Carbon-based nanomaterials have emerged as environmentally friendlier alternatives, with Si-doped carbon quantum dots offering dual-mode up-conversion/down-conversion fluorescence^50^, benzothiazole-functionalized carbon dots achieving 3.5 nM detection^51^, and multifunctional red-emission carbon dots enabling simultaneous detection and cell imaging^52^. Metal-organic frameworks, particularly europium-based MOFs, have provided dual-mode visual sensing capabilities with detection limits around 78 nM^53^.

While these nanomaterial-based methods achieve impressive sensitivity, they suffer from substantial practical limitations that severely constrain routine implementation. The synthesis procedures are invariably complex and time-consuming, requiring meticulous control of multiple parameters including pH, temperature, reaction time, and reducing agent concentrations to achieve the desired particle sizes and optical properties. Batch-to-batch reproducibility remains a persistent challenge, introducing variability that compromises method robustness. The nanosystems often exhibit stability issues under varying analytical conditions, and their performance can be significantly affected by changes in pH, ionic strength, or temperature. Furthermore, complex biological matrices can interfere with nanoparticle surface properties, potentially compromising selectivity. The requirement for specialized equipment and expertise for nanomaterial characterization and the regulatory uncertainties surrounding nanomaterial use in pharmaceutical analysis present additional barriers. Environmental concerns, particularly regarding cadmium-based quantum dots, and relatively long incubation times (10–30 min) further limit their practical appeal for routine quality control applications.

Aptamer-based and biorecognition sensors represent sophisticated molecular recognition approaches offering high selectivity. The TetBBR38S aptamer-based fluorometric method exploits “light-up” fluorescence enhancement of berberine’s intrinsic emission, achieving a detection limit of 0.369 µg/mL with rapid 5-minute analysis for Kampo medicines^54^. DNA-templated silver nanoclusters have enabled ratiometric detection with 10 nM sensitivity, though requiring 30-minute incubation periods^47^. Advanced portable platforms incorporating Bluetooth connectivity and synchronous transport signal technology have achieved 28.32 ng/mL detection limits suitable for on-site analysis of traditional Chinese medicines and environmental samples^55^, while immunoassay-based lateral flow systems have demonstrated utility in feed quality control^56^. However, these biorecognition methods demand expensive oligonucleotides or antibodies, cold chain storage infrastructure, and often require enzymatic digestion steps or specialized expertise that limit their accessibility for routine pharmaceutical analysis in resource-constrained settings.

Additional fluorescence-based approaches have explored aggregation-induced emission phenomena using dicyanodistyrylbenzene derivatives (93 nM detection)^57^ and polythiophene derivatives offering dual fluorometric/colorimetric readout (0.4 µM fluorometric detection)^58^. Functionalized nanoparticle systems, such as SiO₂@NH₂@cyanuric chloride nanoparticles, have achieved exceptional sensitivity of 4.7 nM through electrostatic and π-π stacking interactions that restrict berberine’s intramolecular motion^59^.

The present work represents the first systematic investigation of erythrosin B fluorescence quenching by berberine, providing complete mechanistic understanding absent in previous RRS and absorbance studies. Temperature-dependent Stern-Volmer analysis established static quenching, with bimolecular quenching constants (4.13 × 10^13^ M^− 1^s^− 1^) exceeding diffusion limits by three orders of magnitude. Thermodynamic characterization revealed spontaneous, exothermic complexation (ΔH° = -21.36 kJ mol⁻¹, ΔS° = 33.51 J mol^− 1^K^− 1^, ΔG° = -31.35 kJ mol^− 1^) driven by both enthalpic and entropic contributions. PM3 calculations identified three stabilizing interactions (hydrogen bonding 1.7 Å, electrostatic 3.2 Å, halogen bonding 3.9 Å) with binding energy − 0.184059 Hartree, while Job’s method established 1:1 stoichiometry versus previous ternary 1:1:1 associates. Box-Behnken optimization through 27 experiments yielded a validated model (R² = 0.9682, adequate precision = 26.38) mathematically deriving optimal conditions. The method operates in aqueous medium without organic extraction, achieving superior sustainability using commercially available, inexpensive reagents. Moreover, Visible-region emission (555 nm) minimizes autofluorescence interference versus UV-region detection, while simple 6-minute aqueous chemistry enables high-throughput analysis without complex synthesis or specialized expertise.

## Conclusion

This study successfully developed and validated a novel spectrofluorimetric method for berberine determination based on static quenching of Erythrosin B fluorescence through ion-pair complex formation. Comprehensive mechanistic investigations, including Stern-Volmer analysis, thermodynamic studies, Job’s method, and PM3 quantum mechanical calculations, confirmed ground-state complex formation with 1:1 stoichiometry driven by electrostatic interactions, hydrogen bonding, and halogen bonding. The association constant of 3.10 × 10^5^ M^− 1^ at 298 K and negative Gibbs free energy values demonstrated spontaneous and energetically favorable complexation. Systematic optimization using Box-Behnken design identified optimal conditions (pH 6.4, Erythrosin B 13.0 µg/mL, buffer volume 1.1 mL, incubation time 6.0 min), yielding maximum fluorescence quenching efficiency. The validated method exhibited excellent linearity (0.1–3.0 µg/mL, r^2^ = 0.9997), high sensitivity (LOD = 0.032 µg/mL), satisfactory accuracy (99.83 ± 0.982%), and precision (RSD < 1.4%). Statistical comparison with HPLC reference methods confirmed method equivalence for standardized berberine formulations. Sustainability assessment using AGREE, MoGAPI, CaFRI, and BAGI metrics demonstrated superior environmental performance, with scores of 0.75, 78, 82, and 77.5, respectively, reflecting minimal solvent consumption, low energy requirements, reduced waste generation, and elimination of toxic reagents. The method offers significant advantages over conventional chromatographic techniques, including simplified procedures, reduced analysis time, lower operational costs, and enhanced environmental sustainability. This work represents the first application of Erythrosin B-berberine ion-pair complexation for analytical purposes and provides a practical, cost-effective, and environmentally conscious alternative for routine quality control of berberine-containing dietary supplements and pharmaceutical formulations.

## Supplementary Information

Below is the link to the electronic supplementary material.

Supplementary Material 1

## Data Availability

The data presented in this study are available on request from the corresponding author.
