# Supplementary material for: Sustainable spectrofluorimetric determination of berberine in dietary supplements via Erythrosin B Ion-Pair complexation with mechanistic investigation, Box-Behnken optimization, and green chemistry assessment
Source: Sci Rep. 2026 Feb 3;16:4712. doi: 10.1038/s41598-026-36903-6 (PMC12868707; doi:10.1038/s41598-026-36903-6)
Supplement: Supplementary file 1 — Supplementary Material 1 [file 41598_2026_36903_MOESM1_ESM.docx]

**Sustainable Spectrofluorimetric Determination of Berberine in Dietary Supplements via Erythrosin B Ion-Pair Complexation with Mechanistic Investigation, Box-Behnken Optimization, and Green Chemistry Assessment**

**Humood Al Shmrany ^a^, Ali Alqahtani ^b^,** **Taha Alqahtani ^b^,** **Adil Alshehri ^c^,** **Ahmed A. Almrasy ^d, *^**

^a^ Department of Medical Laboratory, College of Applied Medical Sciences, Prince Sattam bin Abdulaziz University, Alkharj, 11942, Saudi Arabia

^b^ Department of Pharmacology, College of Pharmacy, King Khalid University, Abha, 62529, Saudi Arabia

^c^ Department of Medicine, College of Medicine, King Khalid University, Abha, 62529, Saudi Arabia

^d^ Pharmaceutical Analytical Chemistry Department, Faculty of Pharmacy, Al-Azhar University, Cairo 11751, Egypt

*Corresponding author email address) **Ahmed A. Almrasy**): [ahmedalialmrasy8@gmail.com](mailto:ahmedalialmrasy8@gmail.com)

**Table S1:** Temperature-dependent Stern-Volmer constants, association constants, and thermodynamic parameters for Erythrosin B-berberine complexation.

| Temperature (K) | Ksv (10^5^ M^-1^) | K_a_ (10^5^ M^-1^) | ΔG (kJ mol^−1^) | ΔH (kJ mol^−1^) | ΔS (J mol^−1^ K^−1^) |
| --- | --- | --- | --- | --- | --- |
| 298 | 3.68 | 3.10 | -31.35 | -21.36 | 33.51 |
| 303 | 3.33 | 2.74 | -31.56 |  |  |
| 313 | 2.77 | 2.05 | -31.85 |  |  |

**Table S2:** Box-Behnken experimental design matrix with actual factor levels.

|  |  | Factor 1 | Factor 2 | Factor 3 | Factor 4 |
| --- | --- | --- | --- | --- | --- |
| Std | Run | A:pH | B:Erythrosin B | C:Buffer Volume | D:Incubation time |
|  |  |  | µg/mL | mL | min |
| 24 | 1 | 5.5 | 15 | 1 | 10 |
| 11 | 2 | 3 | 10 | 1 | 10 |
| 2 | 3 | 8 | 5 | 1 | 7 |
| 26 | 4 | 5.5 | 10 | 1 | 7 |
| 7 | 5 | 5.5 | 10 | 0.5 | 10 |
| 25 | 6 | 5.5 | 10 | 1 | 7 |
| 18 | 7 | 8 | 10 | 0.5 | 7 |
| 1 | 8 | 3 | 5 | 1 | 7 |
| 10 | 9 | 8 | 10 | 1 | 4 |
| 9 | 10 | 3 | 10 | 1 | 4 |
| 13 | 11 | 5.5 | 5 | 0.5 | 7 |
| 3 | 12 | 3 | 15 | 1 | 7 |
| 21 | 13 | 5.5 | 5 | 1 | 4 |
| 8 | 14 | 5.5 | 10 | 1.5 | 10 |
| 4 | 15 | 8 | 15 | 1 | 7 |
| 6 | 16 | 5.5 | 10 | 1.5 | 4 |
| 14 | 17 | 5.5 | 15 | 0.5 | 7 |
| 17 | 18 | 3 | 10 | 0.5 | 7 |
| 23 | 19 | 5.5 | 5 | 1 | 10 |
| 27 | 20 | 5.5 | 10 | 1 | 7 |
| 20 | 21 | 8 | 10 | 1.5 | 7 |
| 5 | 22 | 5.5 | 10 | 0.5 | 4 |
| 19 | 23 | 3 | 10 | 1.5 | 7 |
| 22 | 24 | 5.5 | 15 | 1 | 4 |
| 15 | 25 | 5.5 | 5 | 1.5 | 7 |
| 16 | 26 | 5.5 | 15 | 1.5 | 7 |
| 12 | 27 | 8 | 10 | 1 | 10 |

**Table S3:** Model fit statistics for the reduced Box-Behnken design.

| Std. Dev. | 0.1919 |  | R² | 0.9682 |
| --- | --- | --- | --- | --- |
| Mean | 2.83 |  | **Adjusted R²** | 0.9540 |
| C.V. % | 6.78 |  | **Predicted R²** | 0.9322 |
|  |  |  | **Adeq Precision** | 26.3807 |

**Table S4:** Detailed accuracy and precision data for the developed spectrofluorimetric method at individual concentration levels

| Concentration Level | Nominal Conc. (μg/mL) | Repeatability (Intra-day, n=3) | | | Intermediate Precision (Inter-day, n=9) | | |
| --- | --- | --- | --- | --- | --- | --- | --- |
|  |  | Found ± SD (μg/mL) | RSD (%) | Recovery (%) | Found ± SD (μg/mL) | RSD (%) | Recovery (%) |
| Low | 0.5 | 0.498 ± 0.005 | 1.00 | 99.6 | 0.496 ± 0.007 | 1.41 | 99.2 |
| Medium | 1.5 | 1.493 ± 0.014 | 0.94 | 99.5 | 1.491 ± 0.019 | 1.27 | 99.4 |
| High | 2.5 | 2.509 ± 0.025 | 1.00 | 100.4 | 2.506 ± 0.034 | 1.36 | 100.2 |
| Overall |  |  | **0.984** | **99.83** |  | **1.318** | **99.60** |

**Table S5:** Interference study of pharmaceutical excipients on berberine determination.

| Excipient | QE% | SD | Interference |
| --- | --- | --- | --- |
| None (Control) | 67.96 | 1.18 | - |
| Lactose monohydrate | 68.12 | 1.35 | No |
| Microcrystalline cellulose | 67.84 | 1.42 | No |
| Magnesium stearate | 68.31 | 1.28 | No |
| Starch | 67.73 | 1.55 | No |
| Gelatin | 67.91 | 1.33 | No |
| Titanium dioxide | 68.24 | 1.61 | No |
| Silicon dioxide | 67.88 | 1.52 | No |
| Polyvinylpyrrolidone | 68.45 | 1.73 | No |
| Sodium stearyl fumarate | 67.65 | 1.38 | No |

**Table S6:** Cross-reactivity study with structurally related alkaloids and phytochemicals.

| Compound | Class | MW | QE% | SD | Interference |
| --- | --- | --- | --- | --- | --- |
| Berberine (alone) | Protoberberine | 371.81 | 67.96 | 1.18 | - |
| Palmatine | Protoberberine | 352.39 | 92.45 | 2.87 | Yes (36% increase) |
| Coptisine | Protoberberine | 320.32 | 89.73 | 2.73 | Yes (32% increase) |
| Sanguinarine | Benzophenanthridine | 332.34 | 95.18 | 3.18 | Yes (40% increase) |
| Quercetin | Flavonoid | 302.24 | 68.12 | 1.48 | No |
| Rutin | Flavonoid glycoside | 610.52 | 68.17 | 1.55 | No |
| Chlorogenic acid | Phenolic acid | 354.31 | 68.03 | 1.39 | No |

**Table S7:** Comprehensive comparison of fluorescence-based analytical methods for berberine determination

| Method Category | Detection Principle | Linear Range | LOD | Analysis Time | Matrix | Key Advantages | Major Limitations | Ref |
| --- | --- | --- | --- | --- | --- | --- | --- | --- |
| Ion-Association with Xanthene Dyes |  |  |  |  |  |  |  |  |
| RRS with dyes + cinchona alkaloid | Resonance Rayleigh Scattering at λex/λem = 370/452 nm, pH 4.0-5.0 | 0.04-1.2 μM | 1.4 × 10⁻⁸ M (5.2 ng/mL) | 10 min | Tablets | High sensitivity, simple reagents | RRS detection requires specialized equipment, cinchona alkaloid required, no mechanistic studies, no DoE optimization, no thermodynamic data | [41] |
| Ternary ion-associate with eosin/tetraiodofluorescein + cinchona alkaloid | UV-Vis absorbance 536-543 nm, extraction into 1,2-dichloroethane, pH 8.1 | 0.25-1.5 μM (93-560 ng/mL) | ~25 ng/mL | Not specified | Pharmaceuticals | High molar absorptivity, 1:1:1 ternary complex enhances extractability | Organic solvent extraction required, absorbance detection, complex 3-component system, not green, no mechanistic investigation | [42] |
| Supramolecular & Host-Guest Chemistry |  |  |  |  |  |  |  |  |
| ns-Q[10] supramolecular assembly | AIE-based fluorescence enhancement, λex = 297 nm / λem = 405 nm | 45-95 μM | 4.38 μM | Not specified | Human serum | Bioanalytical application, AIE mechanism | Requires specialty ns-Q synthesis, limited availability, high cost, narrow range | [43] |
| CB[6]-based assembly | Turn-off quenching, λex = 320 nm / λem = 410 nm | 0-32.5 μM | 0.15 μM | 7 sec | River/lake water | Rapid response, environmental application | Cucurbituril synthesis required, specialty reagent, limited commercial access | [44] |
| Nanomaterial-Based Methods |  |  |  |  |  |  |  |  |
| CdTe quantum dots | Static fluorescence quenching, λex = 380 nm / λem = 612 nm | 0.025-8.0 μM | 6.0 nM | 10 min | Tablets | Exceptional sensitivity | Cd toxicity concerns, complex synthesis, batch variability, environmental hazard | [45] |
| Au nanoclusters | IFE + aggregation, λex = 329 nm / λem = 566 nm, large Stokes shift (237 nm) | 1.0-100 μM | 75 nM | 2 min | Tablets | Large Stokes shift, rapid analysis | Complex synthesis, precise size control required, reproducibility issues | [46] |
| DNA-templated Ag NCs | Ratiometric fluorescence, λex = 360/590 nm / λem = 560/660 nm | 0.01-1.0 μM | 10 nM | 30 min | Tablets | Ratiometric detection, good selectivity | DNA template synthesis, expensive oligonucleotides, cold storage required | [47] |
| Chitosan/SiO₂-Ag NCs | Fluorescence enhancement, λex = 344 nm / λem = 522 nm | 0.14-16 μM | 30.8 nM | 30 min | Tablets | Enhanced stability, coating protection | Multi-step synthesis, surface modification complexity | [48] |
| Plasmon-enhanced Ag nanocubes + CdTe QDs | PEF with aptamer, λex = 410 nm / λem = 626 nm | 0.1-100 μM | 87.3 nM | 20 min | Tablets, urine | Aptamer selectivity, dual functionality | Complex fabrication, Cd concerns, expensive aptamer | [49] |
| Si-doped carbon QDs | Dual up/down-conversion, λex = 350/790 nm / λem = 452 nm | 0.5-30 μM (down); 0-25 μM (up) | 50 nM | 10 min | Urine | Dual-mode detection, up-conversion capability | Hydrothermal synthesis, complex characterization | [50] |
| Benzothiazole-functionalized C-dots | IFE-based quenching, λex = 330 nm / λem = 425 nm | 10-130 μM | 3.5 nM | 10 min | Tablets, urine | Ultra-high sensitivity, functionalized surface | Complex functionalization, synthesis optimization needed | [51] |
| Red-emission multifunctional C-dots | Dynamic quenching, λex = 474 nm / λem = 618 nm | 10-175 μM | 1.5 μM | Not specified | Cell imaging | Cell imaging capability, red emission | Lower sensitivity, complex synthesis, application-specific | [52] |
| Eu-based MOF | Static quenching via energy transfer, λex = 280 nm / λem = 622 nm | 0.5-320 μM | 78 nM | 15 min | Urine, tablets | Wide linear range, dual-mode sensor | MOF synthesis complexity, ligand preparation required | [53] |
| Aptamer & Biorecognition Sensors |  |  |  |  |  |  |  |  |
| TetBBR38S aptamer | Light-up fluorescence enhancement, λex = 360 nm / λem = 535 nm | 0.78-50 μg/mL | 0.369 μg/mL | 5 min | Kampo medicines | Rapid analysis, molecular recognition | Expensive aptamer, SELEX selection required, cold storage, limited shelf-life | [54] |
| Portable STSHSP platform | IFE with TPDCA probe, λex = 345 nm / λem = 416 nm, Bluetooth connectivity | 0.1-80 μg/mL | 28.32 ng/mL | 1 min | River water, urine, TCM | On-site detection, portable device, rapid | Device cost, probe synthesis, calibration maintenance, specialty equipment | [55] |
| Immunoassay lateral flow | Competitive fluorescence microspheres, UV visualization | Built-in matrix curve | 2.0 mg/kg | 10 min | Animal feed | Visual detection, field-applicable | Lower sensitivity, antibody production, qualitative/semi-quantitative, matrix-specific | [56] |
| Other Fluorescence Approaches |  |  |  |  |  |  |  |  |
| Dicyanodistyrylbenzene AIE | Turn-on via aggregation, λex = 350 nm / λem = 535 nm | 5-35 μM | 93 nM | Short time | River water | AIE mechanism, environmental samples | Probe synthesis required, limited to specific pH/ionic strength | [57] |
| Polythiophene derivative | Dual fluorometric/colorimetric, λex = 420 nm / λem = 530 nm | 1.0-10.0 μM | 0.4 μM (F); 0.27 μM (C) | Immediate | Urine, tablets | Dual readout, immediate response, visual color change | Narrow linear range, polymer synthesis, aggregation sensitivity | [58] |
| SiO₂@NH₂@cyanuric chloride NPs | Light-up via restricted motion, λex = 365 nm / λem = 520 nm | 0.025-2.5 μM | 4.7 nM | Pretreatment required | Bovine serum | Ultra-high sensitivity, bioanalytical | Multi-step NP preparation, salt dehydration pretreatment, complex procedure | [59] |
| Present Method |  |  |  |  |  |  |  |  |
| Erythrosin B fluorescence quenching | Static quenching via ion-pair formation, λex = 530 nm / λem = 555 nm, pH 6.4 | 0.1-3.0 μg/mL (0.27-8.06 μM) | 0.032 μg/mL (86 nM) | 6 min | Dietary supplements | First fluorescence quenching study of Ber-ErB; complete mechanistic characterization; Box-Behnken DoE optimization; aqueous chemistry without organic extraction; commercial reagent; visible emission minimizes interference sensitivity for pharmaceutical QC | Cross-reactivity with quaternary alkaloids; fluorometer required |  |


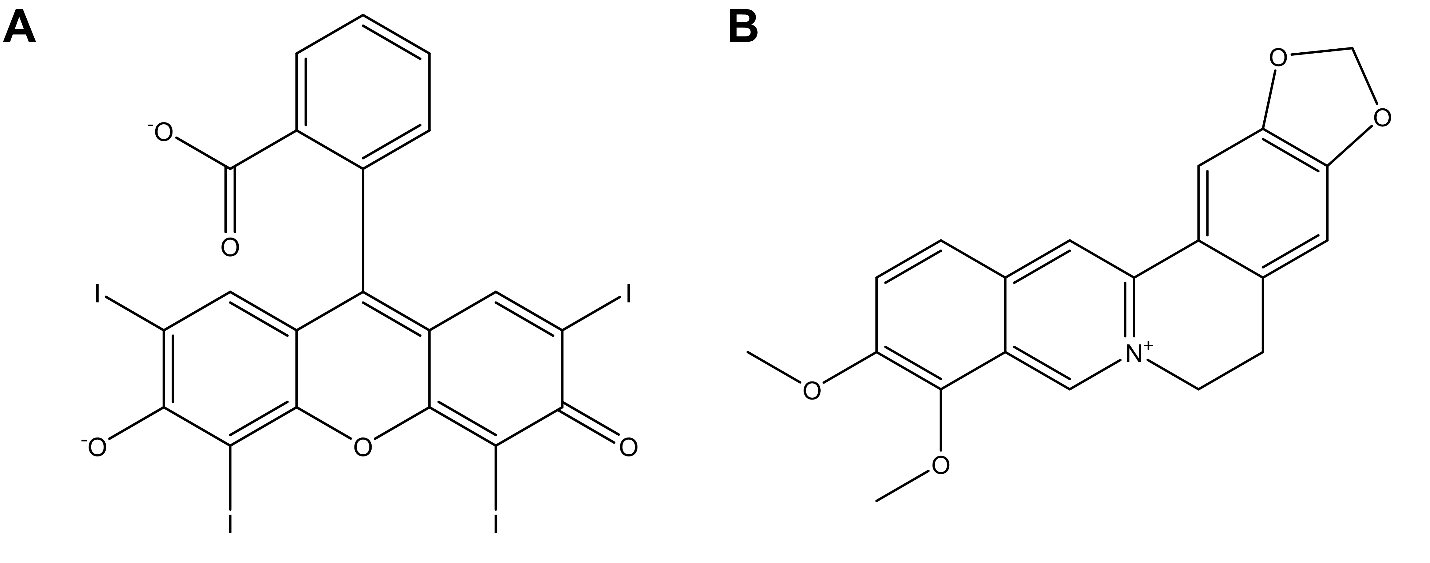


**Fig. S1.** Chemical structures of (A) Erythrosin B showing the xanthene chromophore with four iodine substituents and carboxylate group, and (B) Berberine showing the quaternary nitrogen center and protoberberine skeleton with methoxy groups and dioxole ring. The cationic nature of berberine and dianionic nature of Erythrosin B facilitate electrostatic ion-pair formation.


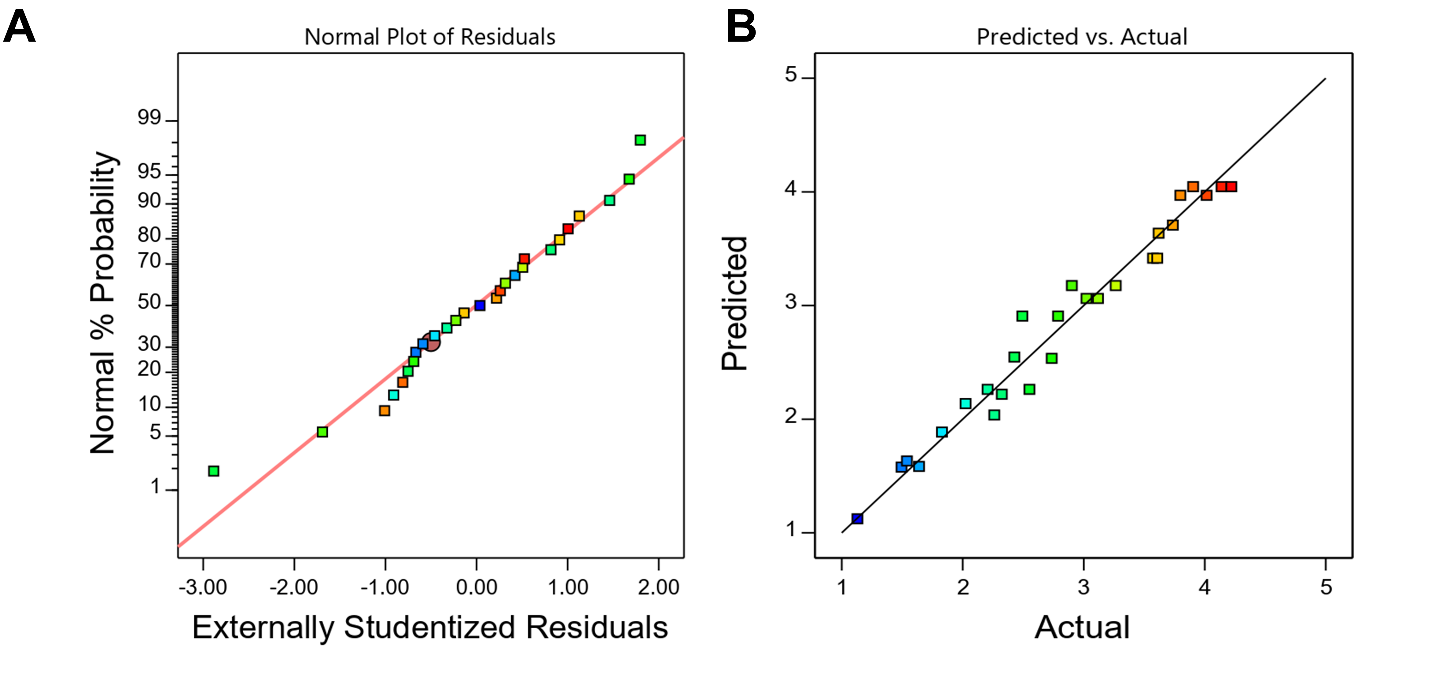


**Fig. S2:** Diagnostic plots for Box-Behnken design model adequacy. (A) Normal probability plot of residuals. (B) Predicted versus actual values plot.


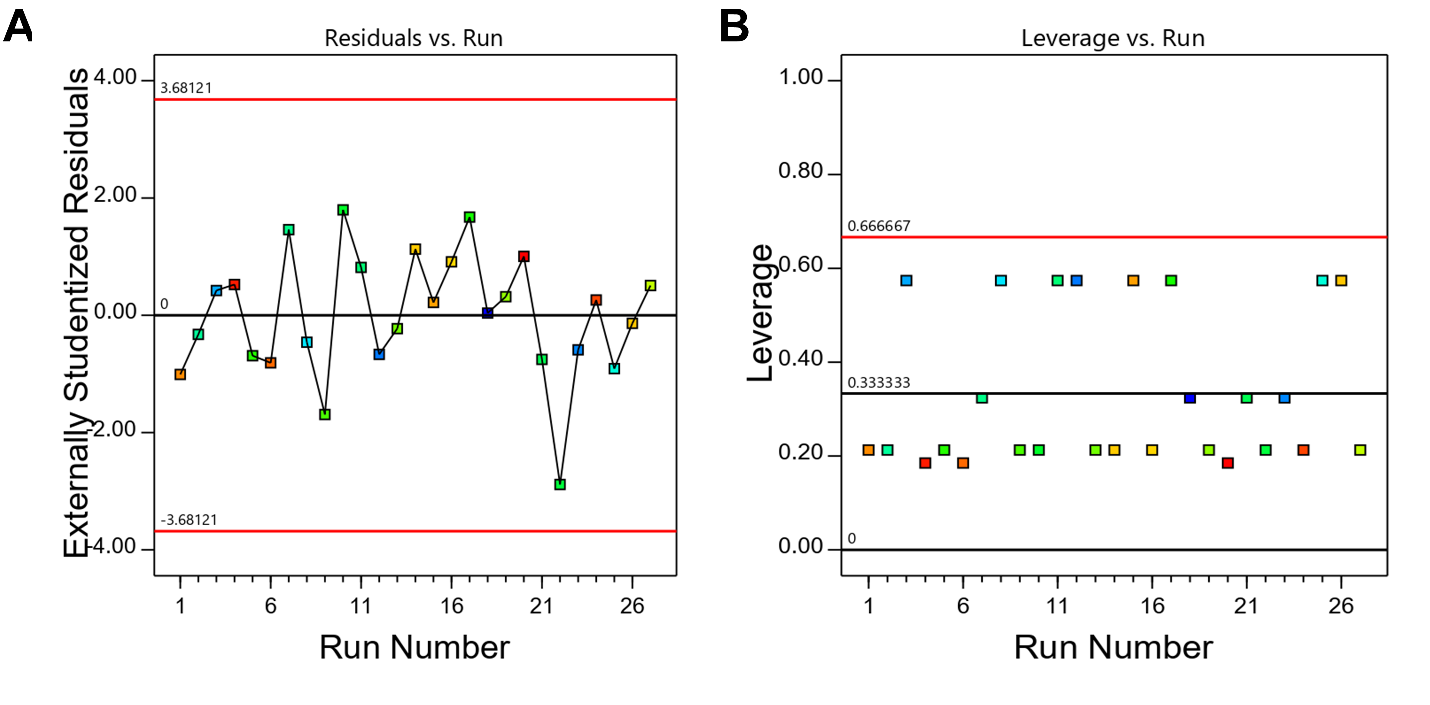


**Fig. S3:** Additional diagnostic plots. (A) Residuals versus run number. (B) Leverage versus run number.


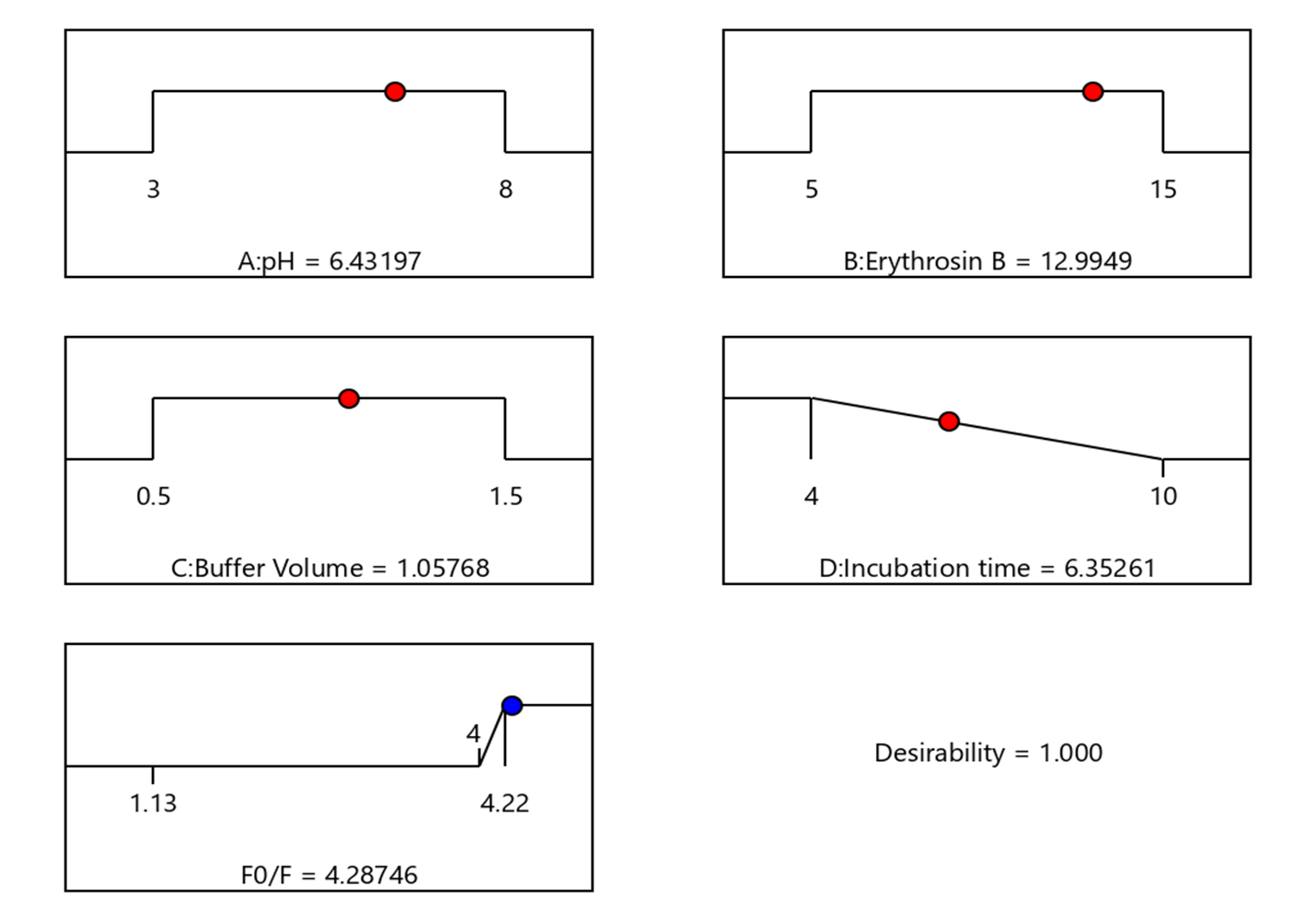


**Fig. S4:** Numerical optimization results showing desirability function approach for identifying conditions maximizing F_0_/F.


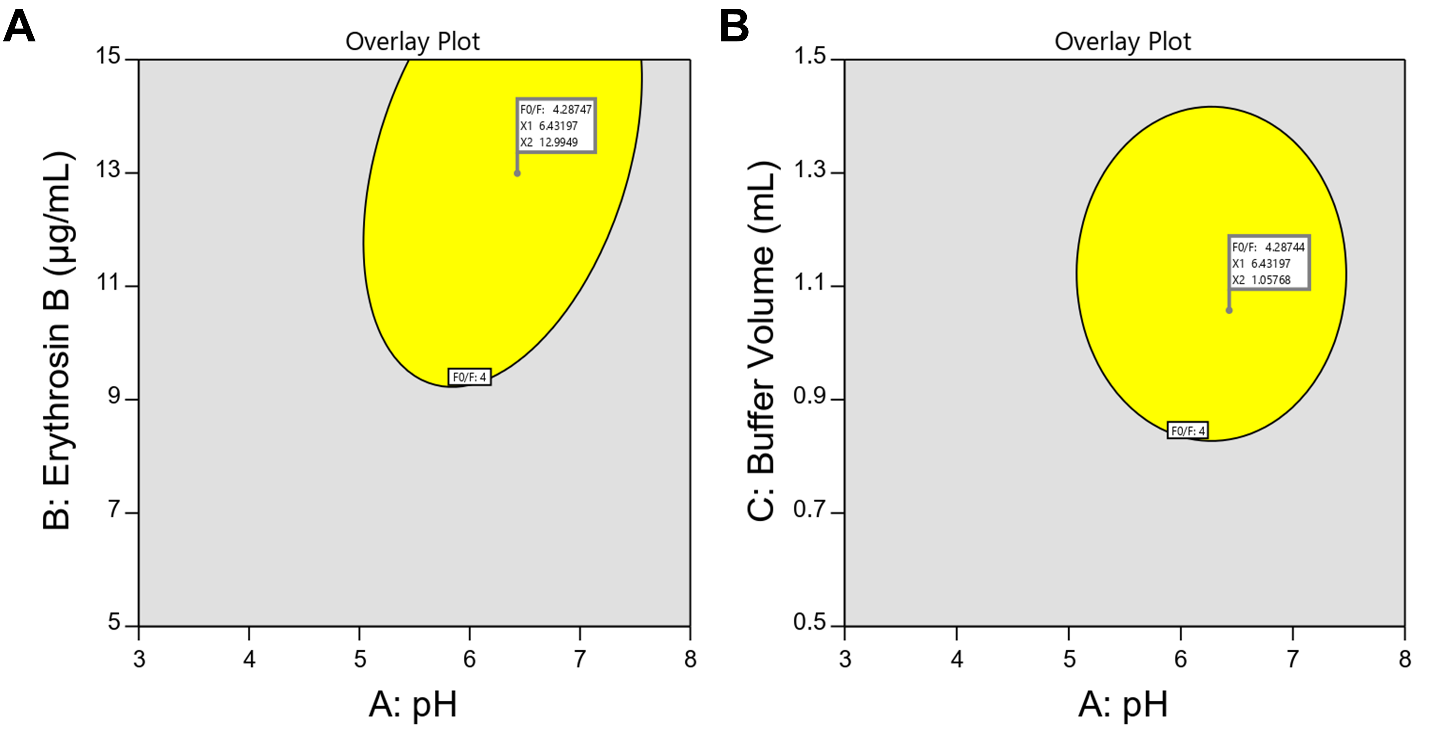


**Fig. S5:** Overlay plot showing feasible region (yellow area) where all optimization criteria are simultaneously satisfied, with optimal conditions indicated.
